# Peptidomimetics and Their Applications for Opioid Peptide Drug Discovery

**DOI:** 10.3390/biom12091241

**Published:** 2022-09-05

**Authors:** Yeon Sun Lee

**Affiliations:** Department of Pharmacology, College of Medicine, University of Arizona, Tucson, AZ 85724, USA; yeon@email.arizona.edu

**Keywords:** opioid receptors, analgesic drugs, bioavailability, peptidomimetic, peptide backbone modifications, locally constrained peptides

## Abstract

Despite various advantages, opioid peptides have been limited in their therapeutic uses due to the main drawbacks in metabolic stability, blood-brain barrier permeability, and bioavailability. Therefore, extensive studies have focused on overcoming the problems and optimizing the therapeutic potential. Currently, numerous peptide-based drugs are being marketed thanks to new synthetic strategies for optimizing metabolism and alternative routes of administration. This tutorial review briefly introduces the history and role of natural opioid peptides and highlights the key findings on their structure-activity relationships for the opioid receptors. It discusses details on opioid peptidomimetics applied to develop therapeutic candidates for the treatment of pain from the pharmacological and structural points of view. The main focus is the current status of various mimetic tools and the successful applications summarized in tables and figures.

## 1. Introduction

Peptidomimetics are synthetically altered peptides with adjusted molecular properties for specific biological or therapeutic applications and have been an important class of drug molecules due to their potential features, high potency, and low toxicity since the term was created first in the late 1970s [1]. Although endogenous opioid peptides such as endorphin (END), enkephalins (ENKs), and dynorphins (DYNs) play various complex roles in the body, they have been shown to have a key role in regulating pathological states of pain as well as in multiple behavioral processes: addiction, reward, sedation, and depression. Their activities in the central nervous system (CNS) have been of particular interest for the treatment of pain, considering the centrally mediated actions of the pain process. Despite their critical roles, the endogenous peptides have limited clinical use due to their lack of drug-like properties: low metabolic stability, poor bioavailability, and low blood-brain barrier (BBB) permeability [2].

To overcome the intrinsic disadvantages and to optimize the therapeutic potential, various strategies have been applied to the design of novel opioid peptidomimetics through in-depth structural analyses and structure–activity relationships (SAR) studies while maintaining key structural features responsible for the biological activity. These, in general, include deletion, addition and/or replacement of certain natural amino acids with non-natural amino acids, and introduction of steric constraints into flexible peptide bonds, which resulted in the discovery of various new opioid peptidomimetic structures with enhanced efficacy and therapeutic potentials.

This article reviews the current development of opioid peptidomimetics from the pharmacological and chemical points of view, which aims toward the development of opioid peptide-derived therapeutics for the treatment of pain. It includes typical mimetic strategies of which the representative molecules with drug-like agonist or antagonist activity are selected and discussed. Emphasis is given to the relatively short length (up to around ten residues) of structures with high receptor selectivity and potency or unique biological profile.

In the following second chapter of this review, opioid receptors and natural peptides are described together with a brief introduction about the discovery and progress. The third chapter covers pharmacological and chemical structural aspects of opioid peptidomimetics. In the former part, pharmacological efforts aimed at developing molecules with enhanced biological activity, receptor selectivity, metabolic stability, BBB permeability, and oral bioavailability are discussed together with successful examples. In the latter part, chemical aspects of opioid peptidomimetics are discussed based on various structural approaches and representative molecules. Starting with the introduction of the well-known “message–address” concept for the opioid receptor-peptide interactions, the part discusses opioid peptidomimetics at MOR, DOR, and KOR for which the relationships to natural peptide scaffolds and activities have been established. The part also outlines useful tools for the opioid peptidomimetic design: local modifications, global restrictions, and secondary structure mimetics. These include the cyclization, *N*/*C*-terminal modifications, peptide linking, local backbone modifications, simple non-natural amino acid replacements, etc. Successful cases are discussed in detail, sometimes with failed ones that are worth introducing. Finally, it details prospective aspects of opioid peptidomimetics as therapeutics aiming to inspire future research.

## 2. Opioid Receptors and Natural Opioid Peptides

Opioid receptors which belong to G-protein coupled receptors are known to be involved in pain modulation, numerous physiological functions, and behavioral effects and are characterized in three subtypes, mu- (MOR), delta- (DOR), and kappa-opioid receptor (KOR) with overall 60–65% high structural homology [3]. The extracellular region has much lower homology, and the differences in the region are responsible for the subtype-selectivity of endogenous opioid peptides [4]. There are three main families of the endogenous opioid peptides, END, ENK, and DYN, which are derived from three different precursor proteins, pro-ENK, pro-DYN, and pro-opiomelanocortin, and prefer to bind at the MOR, DOR, and KOR, respectively, with low selectivity but strong analgesic effects in vivo with milder side effects, unlike morphine [3]. Regardless of the receptor selectivity, all of the endogenous opioid peptides share the same *N*-terminal tetrapeptide sequence (YGGF) that acts as the message part for the receptor, while their *C*-terminal acts as the address part for selectivity (Table 1).

Since their discovery, endogenous peptides, mostly smaller peptides, have been studied extensively to discover a safe analgesic with minimal alkaloid opioid-related adverse effects. Although END was found to be the most potent analgesic due to high MOR selectivity, fewer studies have been done due to the relatively larger size [5]. A SAR study of END showed that *D*Ala substitution at position 2 did not change metabolic stability and biological activity, contrary to the ENK analogs that enhanced the stability and activity [6].

ENKs that are slightly selective for DOR over MOR have been selected as a good scaffold to modify for druggable molecules due to the small size, lower toxicity, and lack of MOR-caused adverse effects. Activation of DOR with an agonist is not strongly analgesic, like that of MOR, but seems to cause less addictive and relatively fewer severe side effects [7,8,9]. *C*^2,5^[*D*Pen^2^, *D*Pen^5^]-ENK (DPDPE), [*D*Ala^2^, *D*Leu^5^]-ENK (DADLE), and [*D*Ser^2^, *D*Leu^5^]-ENK (DSLET) are representative molecules modified from the ENK structure showing enhanced selectivity and affinity with full agonist activity for the DOR [10,11,12]. Interestingly, [*D*Ala^2^, NMePhe^4^, Gly-ol^5^]-ENK (DAMGO) is a highly selective MOR agonist derived from ENK structure as well [13].

DYNs that are formed by cleavage of the precursor prodynorphin contain a high proportion of basic amino acids and exert their analgesic effects primarily through the KOR with slight selectivity over the other subtypes. Activation of the DYN A/KOR system produces similar actions to other opioids but also opposite ones to those of MOR [14]. The KOR appears to be involved in reward, mood state, and cognitive function in the CNS, and its inhibition by an antagonist has recently drawn more attention as a therapeutic target for the treatment of stress-related mood disorders, drug-seeking, and relapse [14,15,16,17,18]. Nonetheless, KOR agonists are still useful if the activity is localized in the peripheral region due to the potential to avoid serious central side effect, dysphoria. The selectivity of DYN for the KOR is known to come from *C*-terminal region including Arg^6^ to Lys^11^, and DYN A-(1-11) is identified as a short fragment that retains similar KOR agonist activity to that of DYN A [19]. Its further modifications led to the development of potent and selective KOR agonists (ex. *N*-benzyl[*D*Pro^10^]-Dyn A-(1-11)) as well as antagonists (ex. *N*-benzyl-c^5,8^[*D*Asp^5^, Dap^8^]-DYN A-(1-11)-NH_2_ (Zyklophin)) [18,20].

Endomorphin (EM)-1 and -2, which are structurally distinct, are atypical endogenous opioid peptides consisting of more constrained amino acid residues (Pro-Trp and Pro-Phe, respectively) in the middle positions and showing higher MOR selectivity over DOR and KOR [21,22,23]. These endogenous peptides are localized in the CNS associated with pain mechanism and in the ending of sensory neurons, and their interactions with the opioid receptors produce clinically effective analgesia, which seems to be dissociated by rewarding, and respiratory depression [23,24,25]. However, their low ability to penetrate the BBB and low metabolic stability prohibit therapeutic uses as analgesics, and therefore significant efforts have been made to improve metabolic stability and to obtain longer lasting antinociceptive effects through various modifications [26,27]. Cyclic EM-1 analogs such as ZH853 (Tyr-c[*D*Lys-Trp-Phe-Glu]-Gly-NH_2_) are recent discoveries showing longer duration of action and effective antinociception in multiple pain models with reduced adverse side effects [28,29,30].

Other naturally occurring peptides are dermorphines (DERs) and deltorphins (DLTs), which are isolated from frog skin and milk peptides and are the most selective for MOR and DOR, respectively [31,32]. The amino acid sequences of these two peptides are quite different from the other endogenous opioids and have been extensively used as a starting point for the development of highly selective opioid peptidomimetics. The *N*-terminal tetrapeptide of DERs, YaFG, is a minimum structure for the MOR activity and its modified analog, [*D*Arg^2^, Lys^4^]-DER (DALDA), is a highly selective MOR agonist but has a limit in crossing the BBB because of multiple positive charges [33]. DLTs are the most selective naturally occurring opioid peptide with a high affinity and potency for the DOR as well as a high BBB penetration rate [34].

## 3. Peptidomimetics for Opioid Receptors

### 3.1. Pharmacological Aspect

#### 3.1.1. Improving Bioactivity, Specificity, and Selectivity for the Opioid Receptors

**Figure 1:**
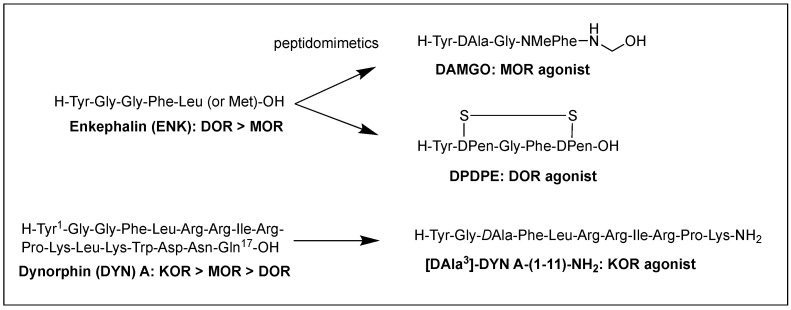
Subtype selective opioid peptidomimetics developed from endogenous opioid peptides.

Structural homology and the natural flexibility of endogenous linear opioid peptides limit their uses for a specific subtype receptor because of the possible interactions with more than one subtype receptors and drastic SAR results caused by subtle structural change. For this reason, selectivity, mostly between MOR and DOR, has been a major problem for the linear opioid peptides, and tremendous efforts have been made to reduce their structural flexibility and thereby enhance the subtype selectivity and potency. Incorporation of a constrained amino acid, configurational change from *L* to *D*, and cyclization of a backbone have been the most useful tool to achieve high selectivity and specificity. Figure 1 displays highly selective opioid peptidomimetics derived from ENK and DYN A for respective subtypes with high potency. DAMGO is a potent and selective MOR agonist to be rendered by introducing an *N*-methyl group into ENK to reduce rotational freedom around the peptide bond (φ/ψ constraint) [13]. Its crystal structure bound to the MOR showed a very similar pose in the binding pocket to those of other small molecule morphinans [35]. DPDPE is the most successful example of cyclization through a disulfide bond that constrains the ENK to an active conformation state for the DOR and has been used the most for scientific purposes [36,37]. It is noteworthy that both selective molecules have originated from less selective ENK. [*D*Ala^3^]-DYN A-(1-11)-NH_2_ is a highly selective KOR agonist with a similar affinity to DYN A-(1-11)-NH_2_ showing how simple substitution can affect receptor selectivity drastically [38].

#### 3.1.2. Transforming Bioactivity from Agonism to Antagonism

While opioid agonists are used for pain relief, antagonists are also substantial in preventing or reversing the opioid agonist effects and intoxication as well as in pharmacological studies to investigate any endogenous opioid regulation. However, there is no opioid antagonist that naturally occurs, and currently available ones have been developed from opioid agonists by structural modifications. In general, agonist versus antagonist behavior of opioid peptides depends on very subtle structural differences, as shown in many cases [3,39,40].

The most effective and generalized structural modification for the conversion into an antagonist is the deletion of an *N*^α^-amino group or its replacement with the other groups such as allyl and hydroxybenzyl [39,41,42]. These modifications similarly affect the three subtypes receptors. Substitution of Tyr^1^ with 3-(2,6-dimethyl-4-hydroxyphenyl)propanoic acid (Dhp) or (2*S*)-2-methyl-3-(2,6-dimethyl-4-hydroxyphenyl)propanoic acid (Mdp) converted opioid agonist peptides to corresponding antagonist peptides with high potency and selectivity [43]. The substitution of position 2 with 1,2,3,4-tetrahydroisoquinoline-3-carboxylic acid (Tic) in ENK and DER analogs converted their respective agonist activities at DOR and MOR to DOR selective antagonists, and NMR analysis indicated a 90-degree arrangement of the two aromatic rings in the *cis*-Tyr-*L*-Tic moiety responsible for the conversion [44]. It was also shown that modifications of EM-2 and morphiceptin (YPFP-NH_2_) at position 3 with *D*Phe or *D-*2-Nal converted the MOR agonist activity to the antagonist activity [40]. Details are discussed in the later part, 3.2. Structural aspects.

#### 3.1.3. Optimizing Drug-like Characteristics including Metabolic Stability, BBB Permeability, and Oral Bioavailability

Clinical application of opioid peptides has been limited due to poor metabolic stability in the body and low ability to cross the BBB [2]. The ability to cross the BBB and reach the CNS to interact with opioid receptors for analgesic effects relies on physicochemical properties including size, lipophilicity, enzymatic stability, hydrogen bonding potential, and other structural features [45]. Physicochemical properties of opioid peptides have been far from optimal and have restricted their oral bioavailability. The nonpeptidic nature of peptidomimetics has the potential to overcome the detrimental therapeutic characteristics and prolong and enhance biological activities at the target site [2,46]. Therefore, various peptidomimetic approaches have been devised to develop molecules with optimized CNS activity, although there are still no definitive alternatives to using the classical analgesics for pain treatment [45,47].

**Figure 2:**
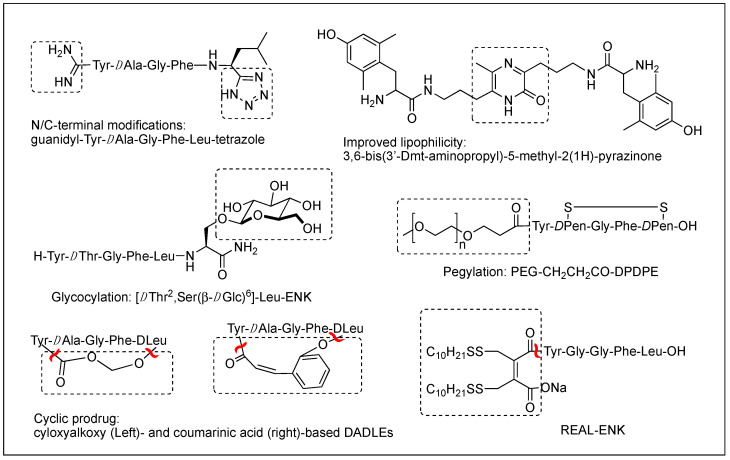
Various modifications to optimize metabolic stability, BBB permeability, and oral bioavailability.

The difficulty of eliciting analgesia with three endogenous opioid peptides is mainly due to the rapid cleavage of the Tyr^1^-Gly^2^ bond, and simple modification of Gly^2^ with *D*Ala^2^ in ENK resulted in long-lasting analgesic effects by avoiding the critical degradation [47,48]. Replacing a peptide bond susceptible to peptidases with a dipeptide isostere such as olefin, ester, and triazole has also been a useful tool to increase peptide stability as well as conjugating with antibodies [47,49,50]. A recent study showed that β-Ala at the *N*-terminus of an ENK-like tetrapeptide amide resulted in longer analgesic effects owing to less accessibility of endopeptidases [51]. Another recent study showed that *N*^α^-guanidinyl group at the *N*-terminus, which mimics the ionic state of the parent peptide, significantly improved the stability and lipophilicity as well as affinity and potency, when combined with *C*-terminal tetrazolate and *D*Ala in guanidyl-Tyr-*D*Ala-Gly-Phe-Leu-tetrazole (Figure 2) [52].

Although metabolic stability has been improved by various structural modifications, the efforts to improve the drug-like property of opioid peptides have been made with limited success for the CNS analgesic effect due to the poor permeability across the BBB. Pharmacological approaches to enhance a peptide delivery to the CNS are to increase biochemical attributes by modifying its structure or conjugating a molecule like lipophilic enhancer, polymer, and antibody. Increasing lipophilicity has been the most useful tool for BBB penetration despite the risk of plasma protein binding [45,53,54]. DPDPE with poor BBB permeability has been modified by methylations, halogenations, *N*^α^-acylation, and *C*-terminal esterification, and the resulting lipophilic analogs have produced significantly increased central analgesic effects as well as BBB permeability [53,55,56,57]. Unlike bis[Tyr-NH]-alkyl peptides, short bivalent analogs linked via an alkyl group, 2′,6′-dimethyl-*L*-tyrosine (Dmt)-substituted analogs produced strong analgesic effects after systemic (s.c. and oral) administrations through the MOR [58]. Among those, pyrazinone-containing analog (Figure 2) was the most potent analgesia in tail flick and hot plate tests. The molecular modeling studies suggested that the bis-Dmt analog contains the identical message and address regions and fits the MOR binding pocket, unlike large dimeric ENK or DER analogs [59]. A study showed that simple *C*-terminal esterification of EM can significantly increase lipophilicity, metabolic stability, and systemic antinociceptive activity after i.c.v. or oral administration, although it provided no explanation on how the change in physicochemical properties are responsible for the side effects [60].

Interestingly, [Dmt^1^]DALDA, a highly polar ligand with a 3^+^ net charge, produced 36 times more potent antinociceptive effects than morphine in mice after subcutaneous (s.c.) administration indicating high BBB transport to the brain [61]. Many studies also demonstrated that hydrophilic peptides could penetrate the cell membrane via absorptive-mediated endocytosis. The common feature was that those peptides contained multiple Arg and/or Lys. E-2078 is also a polycationic DYN A-(1-8) analog internalizing to brain capillaries by the endocytosis [62]. MOR selective DER analogs, H-Tyr-*D*Arg-Phe-Sar/β-Ala-OH, showed potent analgesic effect with low physical and psychological dependence. Their modifications with *N*^α^-amidinoTyr^1^ resulted in the slower onset of the analgesic effect after systemic administration because of the loss of a positive charge and the resulting slow BBB transport [63,64]. Based on the observation, modifying the positive charges might be a useful tool to control the BBB transport and the pharmacological effects in the CNS.

The addition of carbohydrates to a peptide, glycosylation, has also been used to improve BBB penetration and enzymatic resistance while maintaining the biological activity of a parent peptide. The applications for ENKs, DER, DLT, and EM have been successfully performed to enhance central analgesic effects after systemic administration and oral bioavailability [65,66,67,68,69,70,71]. A glycosylated hexapeptide, H-Tyr-*D*Thr-Gly-Phe-Ile-Ser(β-*D*Glc)-NH_2_ (Figure 2), increased metabolic stability and BBB penetration as well as systemic (i.v.) antinociceptive activity, and its further modification with β-lactose and β-melibioside produced highly potent antinociception [67]. The same efforts, glucosylation or galactosylation at the *C*-terminal region, were made for DER and DLT and resulted in a remarkable increase of antinociceptive activity following systemic administration while retaining high in vitro activity as well [72,73]. Thr^4^ glycosylated analog, however, reduced activity dramatically, indicating the important role of the position for opioid activity. Orally active EM-1 analog in which a lactose moiety was linked to the *N*-terminus through a succinamic acid spacer showed a strong antineuropathic effect without producing constipation [71]. A cyclic glycosylated pentapeptide H-Dmt-c^2,4^(-SCH_2_CH_2_S-)[*D*Cys-Aic-DPen]Ser(Glc)-NH_2_ possessing potent mixed MOR agonism/DOR antagonism and poor bioavailability was also successfully modified to afford high antinociceptive efficacy after intraperitoneal (i.p.) administration without acute tolerance [74]. Modeling studies and NMR analysis of glycosylated ENK analogs indicated the modification did not disturb the peptide backbone [75].

Conjugation of polyethylene glycol (PEG) has been used to enhance the therapeutic potential of opioid peptides by reducing enzymatic degradation while maintaining the biological activity of a parent peptide [45]. I.v administration of pegylated DPDPE, PEG-CH_2_-CH_2_-CO-DPDPE (Figure 2), showed an increased analgesic effect despite 176-fold lower binding affinity than DPDPE, which indicated better ability to cross the BBB and undergo hydrolysis in the brain [76]. Nonetheless, there are potential risks of losing activity and brain uptake due to increased molecular size and hydrophilicity or improper applications. To improve the brain uptake, pegylated liposomes as carriers have also been used as a carrier, and glutathione pegylated liposomal formulation of DAMGO was shown to increase and prolong the brain uptake significantly [77].

Prodrugs have been designed to improve the exposure of opioid peptides at target sites, mostly the brain, after systemic administration. Esterification of amine, hydroxyl, or carboxylic acid improves brain uptake by increasing lipophilicity. Likewise, the addition of a Phe residue to DPDPE was shown to increase the BBB permeability as a prodrug [78]. Cyclic prodrugs have also been built using linkers susceptible to esterase hydrolysis for improved delivery characteristics [79,80,81]. For a prodrug, its bioconversion to an active parent peptide within the target sites is the most critical as well as stability in the blood. A study on cyclic prodrugs of DADLE was not successful due to the formation of stable intermediate and the substrate activity for efflux transporters on the BBB nonetheless improved metabolic stability (Figure 2). The enzymatic bioconversion rates were dependent on the chirality of specific amino acids, and therefore alterations in their chirality might be critical for the success of prodrug [82].

Although oral delivery is the most preferred mode of drug administration, the bioavailability of peptides remains low due to degradation and limited absorption in the gastrointestinal tract. The development of oral delivery for opioid peptides requires the same strategies as those applied to optimize the BBB permeability, such as increasing lipophilicity and structural constraint. An amine-modified prodrug of ENK by a reversible aqueous lipidization (REAL) (Figure 2) approach produced prolonged strong oral antinociceptive effects in an inflammatory pain model along with increased metabolic stability, gastrointestinal absorption, and limited CNS-penetration [83]. Thiazole-containing cyclic DAMGO, *N*-terminal amidated-DERs, and phenolic group acylated-DERs are successful examples showing good oral analgesic effect [84,85]. Likewise, *N*^α^-glycosylated EM showed a strong antinociceptive effect after oral administration in the chronic constriction injury (CCI) model but no significant constipation in contrast to morphine [71].

#### 3.1.4. Reducing Opioid Side Effects

There appears to be no substitute for opioids in achieving adequate pain relief in some cases. MOR is primarily responsible for the antinociception but also causes the most undesirable adverse effects, limiting its clinical use. Acute administration causes respiratory depression, constipation, sedation, dizziness, and nausea, and chronic use causes tolerance, dependence, and abuse liability. Despite extensive research in the field, reducing the serious adverse effects of the MOR agonists remains unsolved. The DOR and KOR are also involved in the antinociceptive effect and related side effects, such as convulsion and dysphoria, respectively, to a lesser extent, in vivo. There have been several strategies to design opioid peptidomimetics with reduced adverse effect occurrence [86]. Those are to develop novel ligands that target multiple subtype opioid receptors, interact with receptors peripherally, produce a biased signaling, etc.

##### Multifunctional Ligands

**Figure 3:**
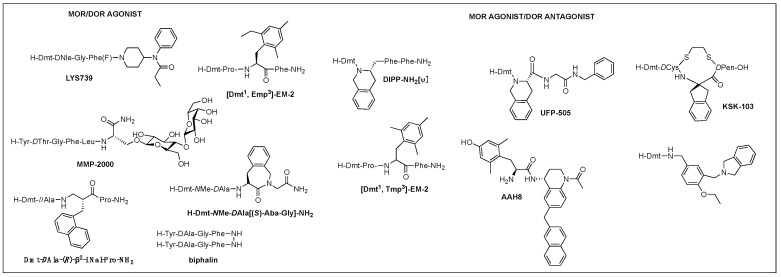
Multifunctional opioid peptidomimetics: MOR/DOR agonist, MOR agonist/DOR antagonist.

Of those, multifunctional opioids that can act on more than one subtype receptor as a single molecule emerged as a promising approach to lessen the opioid-related adverse effects and provide a safer alternative to traditional opioid analgesics [87,88,89]. Mixed agonist activity at the MOR and DOR may bring synergistic antinociception that can reduce adverse effect occurrence by reducing the amount given for the same effect. It was shown that the occupation of DORs by an agonist or antagonist prevented tolerance and physical dependence on morphine [90]. Based on the involvement of DOR in MOR activity proven in numerous studies, such bifunctional ligands with a MOR agonist/DOR antagonist property as AAH8, UFP-505, KSK-103, DIPP-NH_2_[Ψ], [Dmt^1^, 2′,4′,6′-trimethyl phenylalanine (Tmp)^3^]-EM-2, or with a MOR/DOR agonist property as biphalin, [Dmt^1^, 2′-ethyl-6′-methylphenylalanine (Emp)^3^]-EM-2, and LYS739 seemed to be a good approach [74,87,91,92,93] (Figure 3). Biphalin is a homo-bivalent ligand consisting of two tetrapeptides and showing MOR/DOR agonist activities [94]. It produces a substantial antinociceptive effect but causes fewer side effects, suggesting synergistic effects obtained from the activation of both receptors [94,95].

A glycosylated DTLES analog, NMP-2200 (YrGFLS(*O*-β*D*-lactose)-NH_2_) is a centrally active MOR/DOR agonist demonstrated to reduce addiction liability associated with the MOR agonist analgesics through the simultaneous activation of the MOR and DOR [68,96]. Alteration of EM-2 with a Dmt^1^ and an alkylated Phe^3^ (Dmp, Tmp, Emp) led to the bifunctional activities depending on the substitution of Phe^3^: di- and tri-methyl phenylalanine for the DOR antagonism and ethyl methyl phenylalanine for the DOR agonism and increased DOR affinity, yet retained MOR activity, leading to potent MOR/DOR agonism or MOR agonism/DOR antagonim [97]. Metkephamid (H-Tyr-*D*Ala-Gly-NMeMet-NH_2_) is a balanced MOR/DOR agonist producing central analgesic effects after systemic administration in animal models [98,99]. It showed a lesser degree of side effects in extensive clinical tests, indicating DOR activation effect on the CNS. Clinical development, however, was stopped after phase 1 due to unusual side effects, which might be caused by the DOR activation in the CNS. For this reason, MOR agonist/DOR antagonist has been pursued more vigorously than MOR/DOR agonist.

TIPP-NH_2_ was the first to show a mixed MOR agonist/DOR antagonist property, and its further modifications resulted in the discovery of H-Dmt-Tic-[CH_2_NH]Phe-Phe-NH_2_ (DIPP-NH_2_[Ψ]) possessing a balanced MOR agonist/DOR antagonist property and exhibiting a reduced tolerance and dependence upon chronic administration [100]. Several other mixed MOR agonist/DOR antagonists containing a Dmt-Tic pharmacophore were also developed. Intrathecal (i.th.) injection of UFP-505 (H-Dmt-Tic-Gly-NH-Bzl) showed less tolerance in rats than morphine [101]. A 2-aminoindane (Aic)-substituted cyclic analog (KSK-103, Dmt-c(-SCH_2_CH_2_S-)[*D*Cys-Aic-*D*Pen]-OH) with a poor bioavailability also developed fewer tolerance and reward symptoms through the MOR agonist/DOR antagonist activities after *C*-terminal glycosylation [74,102]. Recently, numerous stable structures of MOR agonist/DOR antagonist have been developed showing central analgesic effects after systemic administration, but no in vivo study on the side effects has been reported [92,103,104,105].

Other subtype combinations are MOR/KOR, DOR/KOR, and MOR/DOR/KOR proposed for reducing side effects, but little peptidomimetic structure has been identified [87,106]. Cyclic EM-2 analog, H-Dmt-c[*D*Lys-Phe-2′-MePhe-Asp]-NH_2_ was an agonist for MOR, DOR, and KOR possessing strong antinociceptive effects potentially through the concomitant activation of three receptors [107]. The substitution of β-amino acids has been shown to alter a peptide’s selectivity to a mixed receptor property. Likewise, modifications of a MOR selective morphiceptin with β^2^- or β^3^-amino acid residues resulted in various multifunctional opioid profiles. Dmt-*D*Ala-(*R*)-β^2^-1-Nal-Pro-NH_2_ was a potent MOR/DOR/KOR agonist exhibiting a strong peripheral antinociceptive effect after i.p. and oral administration [108].

##### Localization of the Site: Peripherally Mediated Analgesics

Emerging evidence indicates that opioids also act in the periphery to contribute to analgesic actions, although much less is known about this, compared to the central functions [86,109]. During inflammation, peripheral opioids interact with upregulated opioid receptors in damaged tissue, and the localized interactions can induce therapeutically safe analgesic effects by avoiding undesirable centrally mediated adverse effects [86,109]. The main strategy to localize the interactions in the PNS was to increase the hydrophilicity of the opioid peptides to inhibit the brain uptake. It afforded numerous peripherally acting opioid peptidomimetics with a variety of biological profiles [110].

[β-Pro^2^]-EM-1 is a potent MOR agonist showing peripheral antinociceptive activity after systemic administration along with increased metabolic stability [111]. DAMGO, CR845 (Difelikefalin, H-*D*Phe-*D*Phe-*D*Leu-*D*Lys-[γ-(4-*N*-piperidinyl)amino carboxylic acid]), DALDA, PL017 ([NMePhe^3^, *D*Pro^4^]morphiceptin), CJC-1008, and BW443C (H-Tyr-*D*Arg-Gly-Phe(*p*-NO_2_)-Pro-NH_2_ were shown to produce peripherally localized analgesic effects [112,113,114]. Preliminary clinical studies of BW443C demonstrated peripheral opioid analgesic effects avoiding relevant central effects [115]. CR845, the first approved opioid peptide drug, is in medical use of moderate to severe itching and is under development for the treatment of postoperative and osteoarthritis pain [116]. CJC-1008, a maleimido propionyl group-attached DYN A-(1-13) analog, was designed to promote covalent bond formation with serum albumin and showed a greater peripheral analgesic effect compared to placebo with prolonged activity in Phase II clinical study [113]. Likewise, the conjugation of a biocompatible nano-carrier with a big molecular size also localizes the opioid peptides by blocking the BBB transport [50,86,109].

##### Biased Ligands, etc.

In β-arrestin-2 knockout mice, morphine enhanced analgesia with reduced constipation and respiratory depression [117]. Based on the result, biased ligands that preferentially activate one downstream pathway, G-protein pathway over β-arrestin, have been suggested as a safer, better tolerated, and more efficacious opioid analgesic despite a recent study to the contrary [118,119,120,121]. A study discovered a cyclic peptide, H-Dmt-c[*D*Lys-Phe(*p*-CF_3_)-Phe-Asp]-NH_2_, with high MOR affinity and in vitro functional activity, which turned out to be a G-protein biased MOR agonist [122]. Studies suggested positions 4 and 5 of ENK might be responsible for the biased signaling because the substitutions at the positions resulted in distinctly biased signaling [123,124,125]. Furthermore, positive allosteric modulators were shown to enhance the activities of endogenous opioid peptides, maintain their temporal and spatial action, and potentially limit the adverse effects [126]. Although a few allosteric modulators were identified for opioid receptors and characterized in vitro, their utility in vivo is yet to be determined [127].

### 3.2. Structural Aspect

#### 3.2.1. Conformational Studies and Design from Pharmacophore

The hierarchical approach to peptidomimetics is to (i) identify the conformational structure along with the side chain functional requirements for a target receptor, (ii) reflect the outcomes in building a constrained structure, and (iii) determine the three-dimensional arrangement of the critical side chain and backbone functionalities. Based on this, constrained peptidomimetics in which the peptide scaffold is replaced globally or locally (at a particular amino acid residue or peptide bond) by other organic moieties are designed and synthesized [49,128]. The use of constrained peptidomimetics, new insight into their interactions with opioid receptors, and additional fine-tuning of the constrained structure are critical in developing a novel therapeutic analgesic agent. Recent advances in structural biology using crystallography, molecular docking, molecular dynamics simulations, and NMR spectroscopy provided key insights into the binding modes of opioid peptidomimetics to the receptors. Interestingly, a study using cryo-electron microscopy indicated that the *N*-terminus of DAMGO interact with conserved receptor residue of the morphinan ligand pocket while the *C*-terminus occupies the regions for MOR selectivity [35].

**Figure 4:**
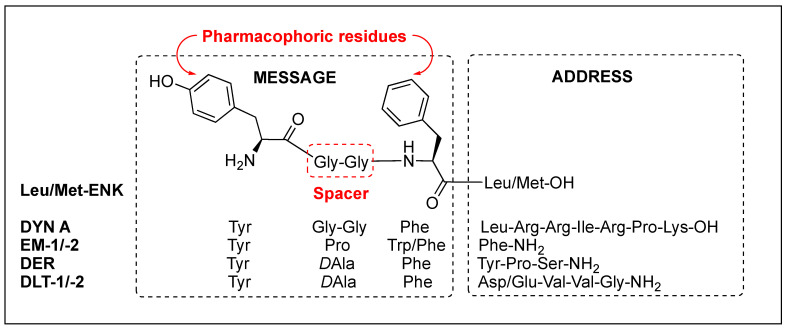
Message and address regions of natural opioid peptides.

Regardless of the sequences, natural opioid peptides contain multiple aromatic amino acids in common as key pharmacophoric residues, and their SARs have long been analyzed based on the message–address concept (Figure 4). The message region of opioid peptides is the *N*-terminal three or tetrapeptide responsible for biological activity, and the address region is the variable structure following the message region responsible for receptor selectivity [129,130,131,132]. In the message region, Tyr and Phe are key pharmacophoric residues, and their *N*^α^-amino, phenolic, and aromatic groups are known to be critical for receptor recognition. Relative orientation and length of the two aromatic rings are also critical for the subtype selectivity. Overall longer backbones are preferred for MOR and shorter ones for DOR. DOR or KOR selectivity over the other subtypes is attributed to the *C*-terminal region, as shown in DLT (the hydrophobic Val^5^-Val^6^ residues) and DYN A (the basic Arg^6^-Lys^11^ residues), respectively. The message–address concept has contributed to the design of highly potent and selective opioid peptides and their modifications for the development of novel opioid peptidomimetics [33,132,133,134].

#### 3.2.2. Peptide Scaffolds as a Starting Point for Mimetics: EMs, DER, Morphiceptin, ENKs, DLTs, and DYN

##### MOR Peptidomimetics

**Figure 5:**
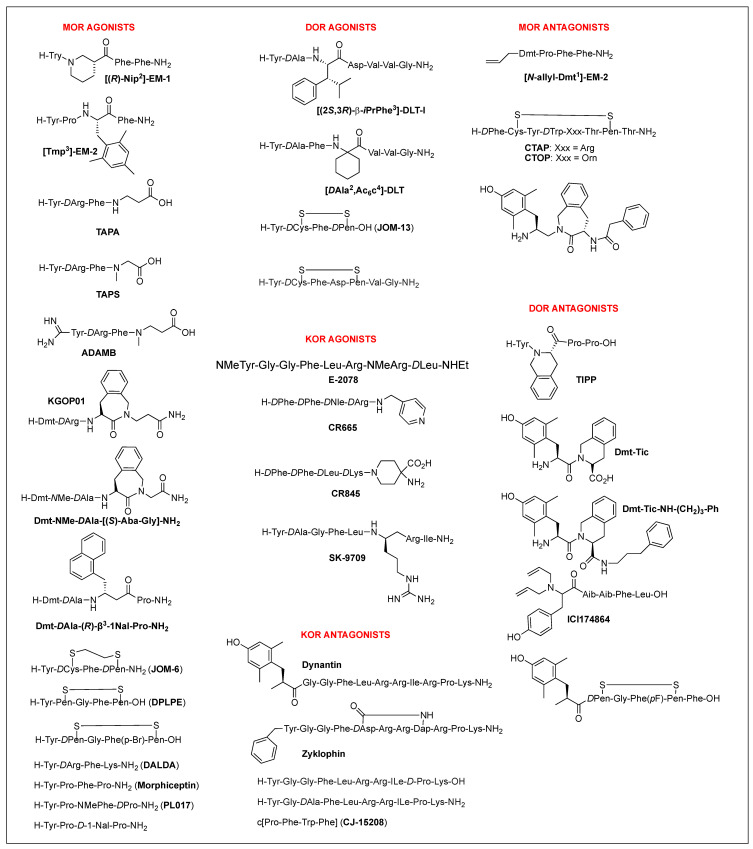
Structures of selective opioid peptidomimetic.

EMs have been suggested to inhibit pain with reduced side effects, particularly the reward effect and respiratory depression [24,25]. Their simple structures, which differ from other endogenous opioids, and high MOR specificity over DOR and KOR have stimulated the structural modifications for overcoming their limits, mainly metabolic instability and low BBB permeability, as therapeutic agents [23,71,107,135,136]. *L*-configuration of Pro^2^, which is a spacer between two aromatic amino acid residues, was shown to be critical, and numerous chemical modifications were focused on the position [23]. NMR analysis and X-ray crystallography revealed that it folded into *cis*-form around the Tyr-Pro amide bond and formed β-turn structure [137,138,139]. Substitution of Pro^2^ with a pseudoproline, β-Pro, C^α,α^-disubstituted glycine, alicyclic β-amino acids, or other substituents was well tolerated, and piperidine-3-carboxylic acid (Nip) derivative, [(R)-Nip^2^]EM-2 showed picomolar range of high affinity for MOR (Figure 5 and Table 2) [133,136,140,141,142,143,144]. Further substitution of Tyr^1^ with Dmt^1^ led to a potent MOR agonist with high stability and analgesic effect, while the selectivity was reduced by increasing DOR affinity [145]. The role of Phe^4^ residue, which is the only distinct residue from morphiceptin, is still not identified, although the majority of results have shown little effect on MOR activity. *C*-terminal modification of Dmt-Pro-Phe-NH-X with various aromatic or aliphatic groups showed the picomolar range of high affinities at MOR as agonists together with weak DOR antagonist activities [146]. Due to its essential role, Tyr^1^ has been the most conserved residue, but several studies showed that its substitution could be tolerated in some cases. c[Tyr-*D*Pro-DTrp-Phe-Gly] contains a Gly bridge for lipophilic property and showed the nanomolar range of binding affinity at the MOR nonetheless lacking the protonable amine [147]. The Phe residue at position 3 is also critical for MOR, and its substitution with sterically hindered Phe residue improved MOR affinities while abolished DOR affinities [148]. The suitable spatial arrangement and conformational restriction of the Phe^3^ seemed to be significant for the MOR activity.

DER is a potent and selective MOR agonist showing a strong analgesic effect after peripheral administration due to slow metabolic degradation [149,150]. The *N*-terminal tetrapeptide is the minimal pharmacophore for the activity, and the *D*-configuration of the second position is critical for the MOR agonist activity [149]. DER tetrapeptide analogs containing *D*Arg^2^ showed very potent and long-lasting antinociceptive activity with various peripheral injections due to high resistance to enzymatic degradation [32,149]. It has been derivatized over the years to yield other highly selective agonists such as [*D*Arg^2^, βAla^4^]-DER-OH (TAPA), [*D*Arg^2^, Sar^4^]-DER-OH (TAPS), *N*-amidino-[*D*Arg^2^, MeβAla^4^]-DER-OH (ADAMB), and DALDA [33,85,151,152]. I.th., i.c.v., or s.c. administration of TAPA produced very potent and long-lasting antinociceptive effects with reduced physical dependence but very low bioavailability [63,151,153]. ADAMB is the most potent DER analog with increased oral bioavailability and slow onset of antinociceptive effect after systemic administration [64,85]. DALDA that contains two positively charged amino acid showed extremely high selectivity for MOR but relatively lower antinociceptive effect after systemic administration due to low BBB permeability [33]. [Dmt^1^]-DALDA displayed very potent antinociceptive effects after systemic administration due to increased resistance to enzymatic degradation, and further modifications resulted in an extremely potent MOR/DOR agonist, Dmt-*N*Me-*D*Ala-[(*S*)-4-amino-1,2,4,5-tetrahydro-2-benzazepine-3-one (Aba)-Gly]-NH_2_, with picomolar potencies [154,155]. Interestingly, these DER analogs containing *D*Arg^2^ showed distinct antinociceptive profiles from the traditional MOR agonists: release of DYN and suppression of the antinociceptive effects by KOR antagonists, as observed in EM-2 [149].

Morphiceptin is a MOR selective agonist identified from β-casein with a unique structure, two Pro residues which allow cis/trans isomerization around the amide bonds between positions 1 and 2, and 3 and 4 [129,156]. The *L*-configuration of Phe^3^ was not critical and its modifications with *D*Phe or *D*-1-Nal improved analgesic effects significantly (>150 folds), and cis-2-aminocyclopentane carboxylic acid (βAc5) substituted analogs improved binding affinities through the formation of MOR favorable *cis*-conformation [40,129,137,157,158,159]. Recent multiple modifications with Dmt^1^, *D*Ala^2^, (*R*)-β^3^-1-Nal^3^ yielded a highly selective and potent MOR agonist, while substitution with (*R*)-β^2^-1-Nal at position 3 produced mixed MOR/DOR/KOR agonist activity showing strong peripheral antinociceptive effects after i.p. or oral administration [108].

Deletion of the *N*^α^-amino group of an opioid agonist or its replacement with the other groups such as allyl and hydroxybenzyl has been utilized for the development of an opioid antagonist with limited success [39,41]. The modifications in Dhp^1^-c[*N*^ε^,*N*^β^-carbonyl-*D*Lys^2^,Dap^5^]-ENK-NH_2_ and [*N*,*N*-bis(*p*-hydroxybenzyl)-Gly]-*D*Arg-Phe-βAla-NH_2_ were less successful due to the diminished MOR selectivity [43,160]. Successful modifications occurred by *N*-allylation in [Dmt^1^]-EMs. The *N*-allylation was the first successful case to convert an alkaloid MOR agonist into an antagonist, which can be a useful tool to investigate the pharmacological role of MOR and the related addiction and abuse [39]. H-*D*Phe-c[Cys-Tyr-*D*Trp-Arg/Orn-Thr-Pen]-Thr-NH_2_ (CTAP/CTOP) is a commonly used, highly selective MOR antagonist derived from somatostatin, and its structural dissimilarity to known opioid peptides made it an attractive starting point. The substitution of *N*-terminal *D*Phe with a constrained analog, *D*Tic, led to the most potent and selective MOR antagonists TCAP and TCOP [161].

##### DOR Peptidomimetics

A potential advantage of targeting DOR in the modulation of pain is reducing opioid-related side effects such as respiratory depression, addiction, and immune functions, and therefore its endogenous ligands, ENKs, have been extensively studied to develop a potent DOR agonist [7,8,9,20,162]. The Phe residue at position 4 was shown to be critical for the DOR activity and selectivity together with Tyr^1^ and *C*-terminal carboxylate [162]. The replacement of Gly at position 2 with *D*Ala reduced DOR selectivity in numerous ENK analogs by increasing MOR activity [163]. However, further replacement of a Leu residue with a *D*Leu in DADLE recovered the DOR selectivity and produced highly potent agonist activity. Interestingly, *C*-terminal truncated [*D*Ala^2^]-ENK tetrapeptide retained the DOR activity, unlike its parent tetrapeptide. Substitutions at the positions other than position 2 with more lipophilic and constrained amino acids occasionally increased binding affinity, selectivity, and potency at the DOR. Despite the critical role of Tyr^1^ and Phe^4^, the first and third amide bonds seemed to be replaceable with other functional groups such as a fluoroalkene without a large loss in the activity [164,165,166]. However, the relative proximity between two aromatic rings at positions 1 and 4 was considered to be critical for the DOR versus MOR selectivity. Based on this, more constrained cyclic analogs were developed using a disulfide bond.

Considering its high selectivity and potency, DPDPE is a major success in DOR peptidomimetic and has been the most commonly used for the studies of biochemical, pharmacological, and physiological properties [20,36,167]. [Des-Gly^3^]-DPDPE (JOM-13, Tyr-c[*D*Cys-Phe-*D*Pen]-OH) designed for additional conformational restriction retained good selectivity and affinity for DOR (Figure 5 and Table 2) [168]. In this scaffold, increasing the ring size by inserting an alkyl group into the disulfide bond resulted in the decrease of DOR activity and selectivity. JOM-6 (Tyr-c^2,4^(SCH_2_CH_2_S)[*D*Cys-Phe-*D*Pen]-NH_2_) with a dithioether ring was a selective MOR agonist. Various modifications at position 4 with constrained amino acids were not successful due to its important role except *p*-alkyl substituted or β-methyl substitution. Interestingly, [Dmt^1^/β-methyl-2′,6′-dimethyl tyrosine (Tmt)^1^, Phe(X)^3^]-DPDPE resulted in highly potent and selective MOR agonists with the improved drug-like property [20]. Conformational analysis of DPDPE and side chain topography led to the development of nonpeptidic or peptidic DOR ligand SL-3111 with 2000-fold selectivity (*K*_i_ = 8.4 nM) for the DOR [169].

DLTs (DLT A, -1 and 2) are highly selective and potent DOR agonists containing *D*-amino acid and anionic residues responsible for the distinctive biological and structural features [31]. Moreover, these peptides are enzymatically stable and permeable to the BBB due to their structural features [34]. Their message region, Tyr-*D*Ala-Phe-, was shown to form a β-turn structure stabilized by the *D*Ala, and the substitution of a Phe residue with a more constrained β-*i*PrPhe residue enhanced the conformation resulting in the exceptional selectivity (30,000 fold) over MOR [170]. The *C*-terminal address region was attributed to the DOR selectivity through the hydrophobic Val^5^-Val^6^ residues, which stabilize an optimal conformation for the binding rather than interact with the receptor [31,171]. The deletion of the anionic character at position 4 resulted in the loss of selectivity due to increased MOR activity. The application of 1-aminocycloalkane-1-carboxylic acids at positions 2, 3, and 4 retained its activity at the DOR, and deletion of the anionic character (Asp^4^) resulted in the enhancement of MOR activity, indicating unfavorable repulsion between negative ion and the MOR receptor [172]. Contrarily, positive net charges were favored for the MOR over DOR, as shown in DALDA [149]. Most cyclic analogs reversed the selectivity due to the marked decrease in DOR binding while some were MOR selective [31]. c[*D*Cys^2^, Pen^5^]-DLT-1 was a highly selective DOR agonist, but c[*D*Cys^2^, Cys^5^]-DLT-1 was a mixed MOR/DOR agonist [173].

*N*,*N*-diallylation of Leu-ENK analog resulted in the discovery of highly selective DOR antagonist (*K*_e_ = 22.0 nM, 227-fold selectivity over MOR), ICI174864 (*N*,*N*-dially-Tyr-Aib-Aib-Phe-Leu-OH) [174]. There are a greater number of specific DOR antagonists than MOR antagonists that exhibit potent bioactivities. H-Tyr-Tic-Phe-Phe-OH (TIPP) and TIP containing a constrained Phe analog, Tic, at position 2 are potent and selective DOR antagonists while *D*Tic-substituted analogs act as a mixed MOR/DOR agonist, showing the configuration-dependent differential effect on selectivity and intrinsic activity [175]. These have been the molecule of interest for SAR studies to investigate the agonist and antagonist properties of the receptor as well as to improve metabolic stability [100,175,176]. Truncation of the *C*-terminal dipeptide led to small dipeptide analogs with diverse biological profiles, DOR agonist or antagonist, or MOR agonist/DOR antagonist. H-Tyr-Tic-NH-CH_2_-CH(Ph)_2_ and H-Dmt-Tic were a selective and potent DOR agonist and antagonist, respectively [100,176]. Simple modification of the *C*-terminal carboxylate of TIPP to the carboxamide produced a mixed MOR agonist/DOR antagonist activity, and further modification with Dmt^1^ resulted in a bifunctional activity in DIPP-NH_2_ with a potent analgesic effect. H-Dmt-Ticψ [CH_2_NH]Phe-Phe-NH_2_ (DIPP-NH_2_[ψ]) designed to reduce chemical degradation via diketopiperazine formation showed more potent i.c.v. analgesic effect in rat tail-flick test with no physical dependence and less tolerance than morphine [100,176,177].

Dmt-Tic is the shortest peptide derived from TIPP possessing a highly selective and potent DOR antagonist activity, and its modification showed a variety of MOR and/or DOR selectivity and agonist and/or antagonist activities [178,179,180]. (*S*,*S*)-isomer of Aba-Dmt moiety, where a Tic was replaced and the backbone direction was reversed, turned out to be a potent MOR antagonist (*K*_e_ = 39 nM) [181].

##### KOR Peptidomimetics

DYN A and its fragments, DYN A-(1-13) and DYN A-(1-11), are highly active at KOR, and the message and address regions are essential for adapting a helical conformation extending from Tyr^1^ and Arg^9^. The address region containing multiple basic amino acid residues was suggested to be responsible for the KOR efficacy. Although the relatively large size of the molecule and the extensive metabolism are more problematic, many studies demonstrated important roles of DYN A and its target receptor, KOR, in pain and other disorders such as depression, anxiety, and drug abuse, and more interest is being focused on the therapeutic potentials of KOR agonists and antagonists [182,183,184].

The low metabolic stability has been improved by various modifications such as *D*-amino acid residue and *N*-alkylated amino acid substitution, *C*-terminal amidation, and peptide backbone reduction. Nonetheless, low selectivity, [NMeTyr^1^, NMeArg^7^, *D*Leu^8^]-DYN A-(1–8)-NHEt (E-2078) were examples of successful modifications to impart metabolic stability in the shortest fragment together with ([*D*Ala^2^, Arg^6^Ψ(CH_2_NH)Arg^7^]-DYN A-(1-8)-NH_2_ (SK-9709) (Figure 5 and Table 2) [185]. E-2078 and SK-9709 showed good antinociceptive effects mainly through the KOR in various pain models after systemic administration, indicating the ability to cross the BBB [186,187]. Highly constrained peptidomimetics of Dyn A-(1-8) with a cyclopropyl methyl normetazocine moiety, which mimics a phenolic ring, a basic nitrogen, and a phenyl ring, were potent KOR agonists with a high affinity and selectivity over MOR and DOR as well as a potent antinociceptive effect [19,188].

A KOR agonist, *D*Phe-*D*Phe-*D*Nle-*D*Arg-NH_2_, was discovered by combinatorial library tool and modified at the *C*-terminus to afford a peripherally acting analog, *D*Phe-*D*Phe-*D*Nle-*D*Arg-NH(4-picolyl) CR665 (FE200665) [189,190,191]. In various pain models, this analog showed potent peripheral analgesic and anti-inflammatory effects [192]. The modification of *D*Arg^4^ with dimethyl-*D*Lys^4^ produced an orally active peripherally restricted KOR agonist, CR845 [116]. It binds to nerves and immune cells in the periphery and blocks pain and inflammation without central opioid side effects.

KOR antagonistic peptides are shown to possess high therapeutic potential in treating drug abuse due to limited duration of action, unlike small molecules with an extremely long duration of action. Therefore, significant efforts have focused on the development of potent and selective antagonists using the DYN A scaffold. *N*-terminal monoalkylation of [*D*-Pro^10^]-DYN A-(1-11) resulted in a marked enhancement in KOR selectivity by decreasing MOR affinity and further *N*,*N*-dialkylation produced an antagonist activity [42,193]. The substitution of Pro at position 3 was also reported to be a highly selective KOR antagonist. Although a few key structural features were identified for the KOR antagonist activity, their applications to DYN A resulted in limited success with weak efficacy, low selectivity, and residual agonist property. Later, numerous peptide antagonists have emerged at the KOR, including the [(2*S*)-Mdp^1^]-DYN A-(1-11)-NH_2_ (Dynantin), [AcPhe^1^,Phe^2^,Phe^3^,Arg^4^,*D*Ala^8^]-DYN A-(1–11)-NH_2_ (Arodyn), c^2,5^[*N*-BzlTyr^1^,*D*Asp^5^,Dap^8^]-DYN A-(1-11)-NH_2_ (Zyklophin), and c[Pro-Phe-Trp-Phe] (CJ-15208) by deleting *N*-terminal free amine group [18,19,43,184,194,195,196]. *N*^α^-benzylation of a cyclic DYN A-(1-11) analog resulted in the discovery of a highly selective KOR antagonist with desirable therapeutic potentials such as systemic activity, proper duration of action, and the BBB penetration in the treatment of diseases such as depression, anxiety, and drug abuse [18,182]. Zyklophin is a selective KOR antagonist with the ability to inhibit the KOR selectively and high potential as a pharmacological tool and a therapeutic agent [18].

**Table 2:** In vitro biological activities of representative opioid peptides and peptidomimetics at MOR, DOR, and KOR *^a^*.

	Binding Affinity, IC_50_ (or *K*_i_) [nM]			Inhibitory Potency, IC_50_ [nM]			Refs.
	MOR	DOR	KOR	MOR (GPI)	DOR (MVD)	KOR (GPI or RVD)	
DAMGO	1.86	345	6090	4.50	32.8	105	[197]
EM-1	0.36	1506	5430	4.03	283	-	[21,39]
EM-2	0.69	9233	5240	6.88	344	-	[21,39]
[(*R*)-Nip^2^]-EM-2	0.04	>1000	-	0.0002 *^b^*	>1000 *^b^*	-	[144,145]
[Tmp^3^]-EM-2	0.44	1400	-	1.90	>10,000	-	[97]
DER	0.76	73.5	>1000	1.07	23.2	-	[149,198]
ADAMB	12.9	>1000	>1000	-	-	-	[85]
DALDA	1.69	19,200	4230	254	781	-	[33,154]
Dmt-DALDA	0.143	2100	22.3	1.41	23.1	-	[33,154]
Dmt-NMe-*D*Ala-[(*S*)-Aba-Gly]-NH_2_	14.8	5.0	>100,000	0.00174 *^c^*	0.016 *^c^*	-	[155]
JOM-6	0.29	24.8	2000	2.93 *^d^*	338 *^d^*	-	[199]
Morphiceptin	79.4	>1000	-	318	4800	-	[159,200]
PL017	2.9	4200	-	21	1250	-	[200]
PL032	5.5	>10,000	-	29	1510	-	[200]
H-Tyr-Pro-*D-*1-Nal-Pro-NH_2_	1.9	>1000	-	9.57	35.4	-	[160
H-Dmt-*D*Ala-(*R*)-β^3^-1-Nal-Pro-NH_2_	0.34	238	1750	7.94	inactive	inactive	[108]
H-Dmt-*D*Ala-(*R*)-β^2^-1-Nal-Pro-NH_2_	0.05	1.04	11.2	3.55	89.1	224	[108]
Met-ENK	9.5	0.91	4440	190	19	-	[201,202]
Leu-ENK	9.43	2.53	8210	35.6	1.73	550	[154,197]
DADLE	13.8	2.06	16,000	8.9	0.73	134	[197]
DPDPE	713	2.72	>15,000	3000	4.14	>10,000	[197]
DPLPE	659	2.80	>15,000	2350	2.77	>10,000	[197]
[Phe(*p*-F)^4^]-DPLPE	1600	0.43	-	740	0.016	-	[203]
JOM-13	51.5	0.74	-	460	4.2		[204]
DSLET	31	3.8	-	360	0.58	-	[205]
DLT-1	2140	0.60	-	2890	0.36	-	[173]
DLT-2	1680	0.73	-	3180	0.67	-	[173]
[(2*S*,3*R*)-β-*i*PrPhe^3^]-DLT-I	63,000	2.14	-	32,500	1.23	-	[170]
c[*D*Cys^2^, Pen^5^]-DLT-1	3760	2.2	-	1100	0.25	-	[173]
c[*D*Cys^2^, Cys^5^]-DLT-1	5.15	0.87	-	2.98	0.23	-	[173]
[DAla^2^,Ac_6_c^4^]-DLT	2.45	0.045	-	558	0.52	-	[172]
β-END	1.0	1.0	52	3.5	2.1	-	[206,207]
biphalin	1.4	2.6	-	8.8	27	-	[208]
DYN A	5.04	2.54	0.23	2.5	22.5	12.6	[209]
DYN A-(1-13)- NH_2_	1.29	4.07	0.15	1.7	78	63.1	[209]
DYN A-(1-11)-NH_2_	1.08	6.99	0.077	7.5	104	0.376	[209,210]
[*D*Ala^3^]-DYN A-(1-11)-NH_2_	67	407	0.36	-	-	2.38	[210]
E-2078	4.51	27.2	1.91	0.3	7.4	2.6	[185]
CR665	4050	20,300	0.24	-	-	0.03 *^c^*	[190]
CR845	-	-	0.32	-	-	0.16 *^c^*	[211]
CTOP	4.3	5600	-	426 *^e^*	-	-	[161]
CTAP	2.1	5310	-	75.8 *^e^*	-	-	[161]
TIPP	1720	1.22	-	inactive	4.80 *^e^*	-	[100]
TIPP-NH_2_	78.8	3.00	-	1700	18.0 *^e^*	-	[100]
DIPP-NH_2_	1.19	0.118	-	18.2	0.209 *^e^*	-	[100]
DIPP-NH_2_[ψ]	0.943	0.447	-	7.71	0.537 *^e^*	-	[100]
Dmt-Tic	1360	1.84	-	inactive	6.55 *^e^*	-	[100]
Dmt-Tic-NH-(CH_2_)_3_-Ph	0.386	0.0871	-	102	1.69 *^e^*	-	[100]
[Pro^3^]-DYN A-(1-11)- NH_2_	5700	8800	2.7	-	-	244 *^e^*	[210]
*N,N*-diallyl-[*D*Pro^10^]-DYN A-(1-11)	31.7	149	3.60	228 *^e^*	-	97 *^e^*	[42]
dynantin	135	354	20.7	925 *^e^*	3220 *^e^*	0.632 *^e^*	[196]
Arodyn	1740	5830	10.0	-	-	-	[212]
Zyklophin	5880	>10,000	30.3	-	-	84 (*K*_B_) *^c^*	[213]

*^a^* The variability in the binding and functional data between various laboratories may be attributed to differences in the synaptosomal preparations, the concentration, type of radioligand, and specific activity used, and the method of reporting binding. Refer to the citations for the details. *^b^* Ca Assay, EC_50_; *^c^* cAMP Assay, EC_50_; *^d^* GTP assay, EC_50_; *^e^* Antagonist mode, *K*_e_; GPI: guinea pig ilem; MVD: mouse vas deferens; RVD: rat vas deferens.

#### 3.2.3. Tools for Mimetics

##### Peptide Backbone Modifications

**Figure 6:**
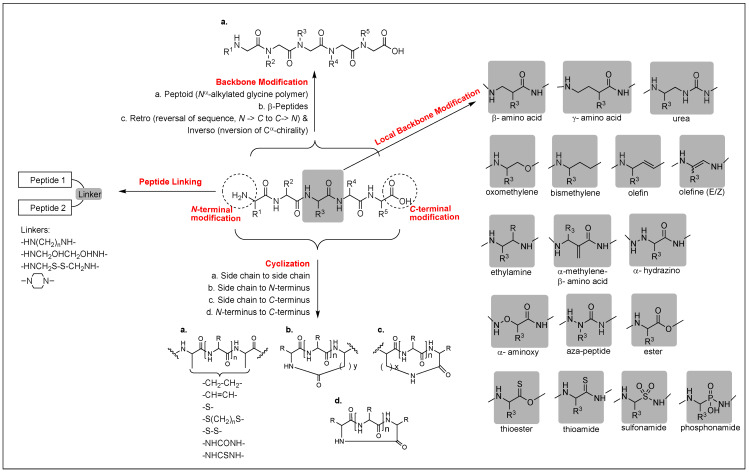
Various tools for peptidomimetics.

Modification of the amide bonds that do not interact with the target is one of the most widely used strategies to optimize the metabolic stability of peptides in vivo, despite the risk of interrupting the conformational profile of the peptides. It is critical to retain the spatial disposition of side chains that are essential for the interaction with the receptors. Peptoids, poly *N*^α^-substituted glycines are α-peptidomimetics, in which the side chains are attached to the amide nitrogen atom rather than to the α-carbon atom, thus causing a loss of the intra-backbone hydrogen bonding and chirality and a formal shift of the side chain position, but improving the resistance to enzymatic hydrolysis and cell permeability [214,215] (Figure 6). These are well suited for the high-throughput library synthesis, and CHIR4531, a peptoid trimer, was the first active opioid peptoid with high MOR affinity (*K*_i_ = 6 nM) discovered from a library of 5000 peptoids [214]. In most cases, however, peptoids or peptoid-peptide hybrid modifications were not successful in the well-known opioid peptides due to resulting conformational changes [216].

β-peptides, poly β-amino acids, possess greater structural diversity and high resistance to the enzymatic degradation due to the additional carbon atom in each amino acid residue, and their biological properties differ from those of parent peptides. Substitution of each amino acid with respective β-amino acids in Leu-ENK, DER, and DLT resulted in a loss of the affinities at MOR and DOR [217]. Other backbone modifications are retro (reversal of backbone), inverso (inversion of chirality of amino acid), and retro-inverso modifications applied to opioid peptides as a tool for structural alteration to improve metabolic stability [218]. These modifications of opioid peptides are not considered to be promising due to the interruption of the spatial disposition of key side chains, despite a few successful cases [218,219]. Overall, studies on backbone modifications of opioid peptides indicated that this approach is not efficient due to resulting structural variability.

##### Cyclization

Cyclization has been a major tool to develop conformationally constrained topographical structures for opioid receptors. Limiting the number of conformations enhances the ability to position the backbone and/or side chains properly for the receptors and improves receptor selectivity and specificity, bioavailability, and metabolic stability [220,221]. Cyclization decreases the number of hydrogen bonding and hydrodynamic radius in a solution but increases lipophilicity, which aids the molecule to cross the BBB to reach the CNS [45]. Constraints of cyclic peptides can be attained through the formation of a covalent bond, such as a lactone, lactam, disulfide, thioether, etc., that spans from the *N*-terminus to *C*-terminus, *N*/*C*-terminus to side chain, or side chain to side chain, which is the most common [128,222,223,224,225] (Figure 6). Typical scaffolds of opioid cyclic peptides are Tyr-c[Xxx-Gly-Phe-Yyy]-OH/NH_2_, Tyr-c[Xxx-Gly-Phe-Yyy], Tyr-c[Xxx-Phe-Phe-Yyy]-OH/NH_2_, Tyr-c[Xxx-Phe-Yyy]-OH/NH_2_, and Tyr-c[Xxx-Phe-Yyy] modified from ENKs, EMs, DER, DLTs, and morphiceptin, where Xxx and Yyy are residues to be consumed for the cyclization through a variety of linkers. However, the cyclization of those small sizes of opioid peptides is not straightforward due to the lack of folding ability in a compact structure.

Due to the essential role of a positively charged nitrogen atom in the receptor recognition, most modifications left Tyr^1^ unaffected. Cyclic EM-1 analogs in which the *N*-terminal amino group was consumed decreased MOR affinities dramatically to the micromolar range, although there were a few exceptional cases, such as c^N,C^[Tyr-*D*Pro-*D*Trp-Phe-Gly] showing moderate binding affinity (Table 3) [147]. Dhp-c^2,5^[*D*Orn-2-Nal-*D*Pro-Gly] was the first cyclic analog without a positively charged nitrogen atom yielding a weak MOR/DOR antagonist [41]. SAR studies on cyclic EM-2 analogs indicated optimal ring size for MOR affinity and selectivity to be 14 while ENK preferred to be 18 [226,227]. I.c.v. and i.p. administrations of cyclic EM-2 analogs (Tyr-c^2,5^[*D*Lys-Phe(F/2,4-difluoro)-Tyr-Gly]) exhibited potent analgesic effects indicating the potential brain uptake, and the structural analyses using NMR spectroscopy suggested that a trans conformation of the backbone between positions 2 and 3 is critical for affinity, selectivity, and functional activity at the MOR. An extended structure of EM-1, ZH853 (Tyr-c[*D*Lys-Trp-Phe-Glu]-Gly-NH_2_) is a recent success showing high potential as a therapeutic agent for various pain states, including neuropathic pain [28,30,228]. Another EM analog, Tyr-c^2,5^(-SCH_2_S-)[*D*Cys-Phe-2-Nal-Cys]-NH_2_ showed a MOR/KOR agonist and DOR partial agonist/antagonist profile with a similar affinity at MOR and DOR [229]. Enhanced KOR activity was observed in a series of cyclic EM pentapeptides, Dmt-c[*D*Lys-Xxx-Yyy-Asp]-NH_2_, in which Xxx and Yyy are Phe or NMePhe [195].

Interestingly, the substitution of a Tyr with a Dmt in cyclic peptides did not affect affinities, unlike linear peptides, but still increased the metabolic stability. Oral administration of a cyclic morphiceptin analog, Dmt-c[*D*Lys-Phe-*D*Pro-Asp]-NH_2_ (P-317), inhibited GI motility and produced a potent and long-lasting MOR-medicated antinociceptive effect due to enhanced enzymatic resistance and anti-diarrhoeal activity [230,231]. The small cyclic tetrapeptide, Tyr-c^2,4^[*D*Lys-Phe-Ala], has the picomolar range of potency for MOR (0.11 nM) and DOR (0.54 nM) in the GPI and MVD assays, and its modification with Asp^4^ significantly reduced both activities, indicating the incompatible effect of the negative charge on both receptors [232]. Interestingly, Tyr-c[*D*Lys-Phe-Asp]-NH_2_, in which the side chain of Asp^4^ was consumed for the cyclization, was a highly potent and selective MOR agonist [227].

Driven by the success of DPDPE, many modifications have been performed mainly to replace a disulfide bond susceptible to enzymatic degradation and nucleophilic, with a stable bond, such as a rigid olefin bond (Figure 6). Both *cis* and *trans* olefin analogs, Tyr-c^2,5^(-*trans*/*cis*CH=CH-)[*D*Ala-Gly-Phe-*D*Ala]-OH, were potent MOR/DOR agonists with a slight selectivity for DOR [224]. A methylamine-bridged ENK analog, Tyr-c[(N_β_CH_3_)-D-A_2_pr-Gly-Phe-NHCH_2_CH_2_-], was a potent MOR/DOR agonist showing substantial antinociceptive effects (ED_50_ = 27 ng) [233]. Lanthionine (thioether) bridge-substituted analogs, Tyr-c^2,5^(-S-)[*D*Ala-Gly-Phe-*D*/*L*Ala]-OH, were also potent MOR/DOR agonists with a subnanomolar analgesic potency (ED_50_ = 0.0015 and 0.0018 nM, respectively) [223]. A series of cyclic ENKs with a urea bridge showed mixed MOR/DOR agonist activity. Tyr-c^2,5^(-NHCONH-)[*D*Lys-Gly-Phe-Dap]-NH_2_ was a very potent MOR/DOR agonist with the subnanomolar range of EC_50_ (0.21 and 0.65 nM in the GPI and MVD, respectively) and ED_50_ (0.0792 and 0.3526 nM in the hot-plate and tale-flick tests, respectively, i.c.v. in rats) [226,234]. Cyclic biphalin analogs through various linkers such as xylene were highly potent MOR/DOR agonists with prolonged analgesic effects after systemic administration [235,236]. Interestingly, a *C*-terminal-modified cyclic biphalin analog, c^2,2′^(Dmt-*D*Cys-Gly-Phe)_2_-cystamine, was a very potent MOR/DOR/KOR agonist with the sub-nanomolar affinities and the same high efficacies with DAMGO, DADLE, and salvinorin A at MOR, DOR, and KOR, respectively [237].

DYN A was cyclized at various positions due to a relatively longer peptide chain, and the positioning and stereochemistry of the cyclization impacted KOR affinity and selectivity. c^N,5^[Trp^3^,Trp^4^,Glu^5^]-DYN A (1–11)-NH_2_ was the first cyclic KOR antagonist lacking the *N*-terminal amino group but retained the nanomolar affinity [238]. Two isomers of c^2,5^(-CH=CH-)[Ala^2^,Ala^5^]Dyn A (1–11)-NH_2_ exhibited a high KOR affinity but similar selectivity to DYN A-(1–11)-NH_2_ [239]. Incorporation of *D*Ala at position 3 of c^2,5^[*D*Asp^2^,Dap^5^]-Dyn A (1–11)-NH_2_ resulted in the increase of KOR selectivity over MOR and DOR [195]. Two isomers of c^5,8^(-CH=CH-)[Ala^5^,Ala^8^]Dyn A (1–11)-NH_2_ having the address region in a cycle significantly increased KOR selectivity compared to Dyn A (1–11)-NH_2_ [239]. The cyclization of a partial KOR agonist, [BzlTyr^1^]-DYN A-(1-11)-NH_2_, at the same positions 5 and 8 through a lactam ring resulted in the development of a highly selective KOR antagonist, zyklophin, capable of crossing the BBB [18,194,213].

##### *N*- and/or *C*-Terminal Modifications: Amide (-NHMe, -NHMe, -NMe_2_), Ester (-OMe, -OEt, -OBu), Hydroxy (-OH), or Hydrazide (-NH-NH_2_)

In addition to the optimization of enzymatic stability, modifications of the *N*- and *C*-terminus of opioid peptides affect functional activity and receptor selectivity, respectively, based on the message–address concept [129,132]. Due to the essential role, *N*-terminal modifications were limited to the trials converting functional activity, from agonism to antagonism, while *C*-terminal modifications were performed frequently to optimize the selectivity and physicochemical property or conjugate with other pharmacophoric structure [39,43,93,245]. Transformations of the *C*-terminal carboxylate into a carboxamide or a carboxylate ester resulted in the reduction of DOR selectivity in ENK and DPDPE due to an enhanced MOR activity [57,167]. The attachment of alkyl urea moiety (-NHCH_2_CH_2_NH(C=O)NH_2_) at the *C*-terminus of ENK retained the same activity at MOR and DOR [246]. Various *C*-terminal amidations of TIPP transformed the selective DOR antagonist into a mixed MOR agonist/DOR antagonist [175]. Simple *C*-terminal modifications of EMs to an ester or a hydrazide showed significant analgesic effects after central (i.c.v.) and peripheral (s.c.) administrations with the reduced tolerance, cardiovascular, and constipation [60]. There was no explanation of how physicochemical properties modulated side effects. A recent study showed *C*-terminal amidated ENK reduced β-arrestin recruitment efficacies at MOR and KOR [247]. The role of the *C*-terminus is still not clear, due to the inconsistent results [60]. Further modification of Phe^4^ to -NH-X- (aromatic, heteroaromatic, and aliphatic groups) resulted in the loss of MOR activity [23].

Deletion and modification of the *N*-terminal amino group alter opioid agonist activities to antagonist activities as discussed in Section 3.1.2 [39,43]. Interestingly, incorporation of lipoamino acids to the *N*-terminus of EM retained MOR agonist activity and afforded a strong antinociceptive effect without causing constipation after oral administration in CCI rats [71]. Cationization of the *N*-terminus of morphiceptin and EM through guanidinylation (NH_2_-C(=NH_2_)-NH-) increased the brain uptake and metabolic stability, although decreased affinity at MOR occurred [71]. *N*-guanidinylated cyclic ureido DLT analogs exhibited the balanced nanomolar affinities at the MOR and DOR, and high enzymatic stability that mediates clinically relevant analgesia [248]. A recent study showed that modifications of the *N*- and *C*-terminus with a guanidine and a tetrazole could improve metabolic stability (t_1/2_ > 6 h) and lipophilicity (clogD = 0.44) without affecting their activities at MOR and DOR [52]. ADAMB is a good example to show long-lasting activity and strong oral antinociceptive activity after the substitutions at the *N*-terminus and position 4 [85]. Recent studies suggested that *C*-terminal modifications can regulate β-arrestin 2 recruitment and thus reduce MOR related side effects [123,124].

##### Peptide Linking

As shown in biphalin, linking two opioid peptides increases potency, efficacy, and bioavailability compared to their corresponding monomeric counterparts in the cases of receptor dimerization or cross-linking of neighboring receptors by triggering receptor-receptor interactions [94,249]. Dual agonist activity at MOR and DOR results in a synergistic antinociceptive effect that is advantageous in reducing dosage, thereby attenuating related side effects. Based on this advantage, peptide linking has been widely used by connecting two address regions through various linkers while retaining two message regions [94,235,250]. Biphalin showed a similar trend on modifications to ENK, and substitutions of Phe^4^ with Phe(X) and Nal residues affected its activity positively together with the linker variation. It was shown that analogs with a hydrophilic linker such as butanediol increased affinity at KOR whereas a piperazine linker produced a balanced high affinity for MOR and DOR [94]. [Dmt^1^]DALDA-NH-CH_2_-CH_2_-NH-TICP(ψ), in which two structural moieties were linked through an ethylenediamine, was a hetero-bivalent ligand displaying MOR agonist/DOR antagonist profile with the nanomolar binding affinities at both receptors [251]. Bitopic ligands, a new class of ligands that simultaneously target orthosteric and allosteric sites, are an extension of the bivalent ligand concept, and no bitopic opioid ligand has been identified yet. However, considering positive allosteric interactions between MOR and DOR, bivalent ligands may act as a bitopic ligand [252].

##### Local Backbone Modifications

Peptide backbone is defined by the φ, ψ, ω, and χ torsional angles [128]. Altering a peptide bond using various non-natural amino acids or isosteres has been widely used to amplify conformational constraints of the dihedral angles and thereby to reduce the rotational freedom of those side chains directly involved in the biological interaction (Figure 6).

Most modifications of ENK using alkylurea, trifluoroethylamine, oxymethylene, olefin, and bismethylene isosteres were not successful except in a few cases [57,253,254,255]. Among them, olefine bonds from α,β-unsaturated α-amino acids conserve the planarity of the peptide bond but no H-bonding because of the deletion of an oxygen atom, an acceptor. A fluoroalkene substituted-ENK, [Tyr^1^-ψ[(Z)CF=CH]-Gly^2^]-Leu-ENK, was a successful case increasing the stability as an orally active CNS-distributed peptide probe [166,256]. Its modifications at the *C*-terminus reduced β-arrestin recruitment through the DOR and MOR while retaining affinity and cAMP potency [124]. Another DOR analogs, [Gly^2^-ψ[(*Z*)CF=CH]-Phe^3^]-Leu-ENK, [Gly^2^-ψ[C(=O]O]-Phe^3^]-Leu-ENK, [Gly^2^-ψ[C(=S)NH]-Phe^3^]-Leu-ENK, retained the agonist activity, indicating the amide bonds can be replaced by a fluoroalkene, an ester, a thioamide possessing high dipole moment and rotational barrier without a substantial loss of activity [165]. Positional scanning of the Leu-ENK with an ester bond and *N*-methylation demonstrated that the fourth amide bond is replaceable [164]. The SAR results of two analogs, [Gly^4^-ψ[C(=O]O]-Leu^5^]-ENK and [NMeLeu^5^]-ENK indicated that an H-bonding acceptor is critical [164]. Trials to enhance the systemic analgesic effect of [Dmt^1^]-DPDPE using oxymethylene (-C-O-), trans-double bond (-C=C-), or bismethylene (-C-C-) isosteres were not successful [57]. β-sulfonamide analog of EM-2 in which Pro^2^ was replaced with 3-pyrrolidinemethanesulphonic acid (βPrs) retained a high affinity and selectivity at MOR (*K*_i_ = 19 nM) [140]. Recently, aza-pipecolyl (azaPip) substituted EM and morphiceptin analogs were developed to study the conformational requirements, whether *cis*-amide geometry and β-turn conformation are critical for the receptor interactions [257]. Its application in H-Dmt-azaPip-*D*Phe-Pro-NH_2_ improved potency and affinity for MOR and DOR, indicating the requisite for natural Tyr-Pro *cis* form for the receptor binding [257].

**Figure 7:**
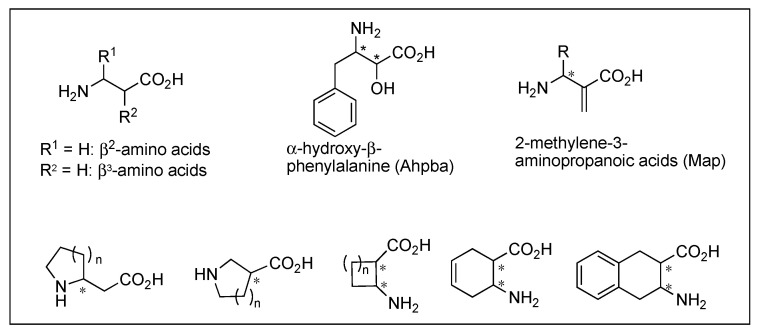
Structures of β-amino acids (upper) and alicyclic β-amino acids (lower). * Chiral.

As a local variation of parent amide backbone, β-amino acids, γ-amino acids, β-dipeptidomimetics, and the other conformationally constrained non-natural amino acids (Figure 7) have been simply applied to opioid peptides [51,258]. Insertion of one (β-amino acids) or two additional carbon atoms (γ-amino acids) into a peptide backbone changed the conformational structure, afforded the structural diversity by increasing diastereomers, and enhanced metabolic stability for prolonged in vivo effect [258]. Incorporation of cyclic amino acids further produced successful peptidomimetics for degradation resistance and restricted conformation. For these reasons, many studies have focused on incorporating a variety of β-, γ-, or cyclic β-amino acids into opioid peptides.

Position 2 of EM has been modified successfully without damaging the affinity but improving the enzymatic hydrolysis resistance despite the reduction of MOR selectivity over DOR [111,136,142,240,259]. Likewise, many studies showed that the substitutions of various β-amino acids including acyclic β-amino acids into EMs were well tolerated at position 2, unlike the other positions [136,141,143]. Substitution of a Pro residue with β^2^-(*R*)-Pro or β^3^-(*S*)-Pro increased or retained affinity at the MOR and exhibited good resistance to the proteolytic enzyme and effective peripheral antinociception [111,260]. Dmt-(1*S*,2*R*)Achc-Phe-Phe(*p*-F)-NH_2_, in which *cis*-2-aminocyclohexane carboxylic acid (Achc) was substituted at position 2 was a potent MOR agonist (EC_50_ = 51 nM) with high efficacy (176% compared to DAMGO) and enhanced proteolytic stability (t_1/2_ > 20 h in rat brain membrane) [136]. Incorporation of β^2^-, β^3^-hPhe, or β^3^-hTic at positions 3 and 4 resulted in the decrease of MOR selectivity by increasing DOR activity while retaining MOR activity, and NMR and molecular modeling studies indicated their predominant turn structures [261,262]. A series of more constrained β-amino acids, 2-methylene-3-aminopropanoic acids (MAP), were incorporated into positions 3 and 4 of EM [263]. H-Tyr-Pro-Trp-(2-Furyl)Map-NH_2_ was a very potent MOR agonist with the picomolar range of binding affinity (*K*_i_ = 0.22 nM), 430-fold increased agonist activity (EC_50_ = 0.0334 nM, E_max_ = 97%) in the cAMP test, high metabolic stability, and enhanced antinociceptive activity in the mice tail-flick test [263]. β-dipeptidomimetics (-β^3^hPhe-Tbac-, -β^3^hPhe-Tia-, -β^3^hPhe-β^2^hPhe-, -β^3^hPhe-β^3^hPhe-, -β^2^hPhe-β^2^hPhe-) replacing the native backbone of Phe^3^-Phe^4^ residue in EM-2 retained MOR affinity due to the prevalent extended conformation [262]. Substitution of a Phe residue with chiral α-hydroxy-β-phenylalanine (AHPBA, βAtc) in EMs and [Dmt^1^,Tic^2^]-EMs retained the biological activities of the parent peptides and improved half-life slightly in most cases [264]. Among those, Dmt-Tic-(2*R*,3*S*)AHPBA-Phe-NH_2_ significantly increased the half-life (>2 h) and retained a strong antinociception (ED_50_ = 2.0 nM, i.c.v.) without causing a motor effect and acute tolerance due to the potential role of DOR antagonist involved.

In morphiceptin analogs that are similar to the structure of EMs, incorporation of *cis*-2-aminocyclopentane carboxylic acid (*cis*-2-Acpc) into position 2 retained the activity and selectivity of parent peptides for MOR due to enough separation of Tyr^1^ and Phe^3^[129,141,157]. Contrarily, the incorporation of *cis*-2-Achc into DER lost MOR activity, indicating its unfavorable conformation for the receptor and distinct receptor interaction from morphiceptin [265]. In multiply modified analogs, the substitution of β^2^- or β^3^-amino acids affected the selectivity depending on their configurations and resulted in the development of an agonist, H-Dmt-*D*Ala-β^2^-(*R*)-1-Nal-Pro-NH_2_, for MOR, DOR, and KOR (*K*_i_ = 0.05, 1.04, and 11.2 nM, respectively) with high metabolic stability (t_1/2_ > 50 h) [108]. Incorporations of β^3^-homo amino acids into the message region of DLT decreased binding affinity at DOR except positions 2 and 3 [217].

**Figure 8:**
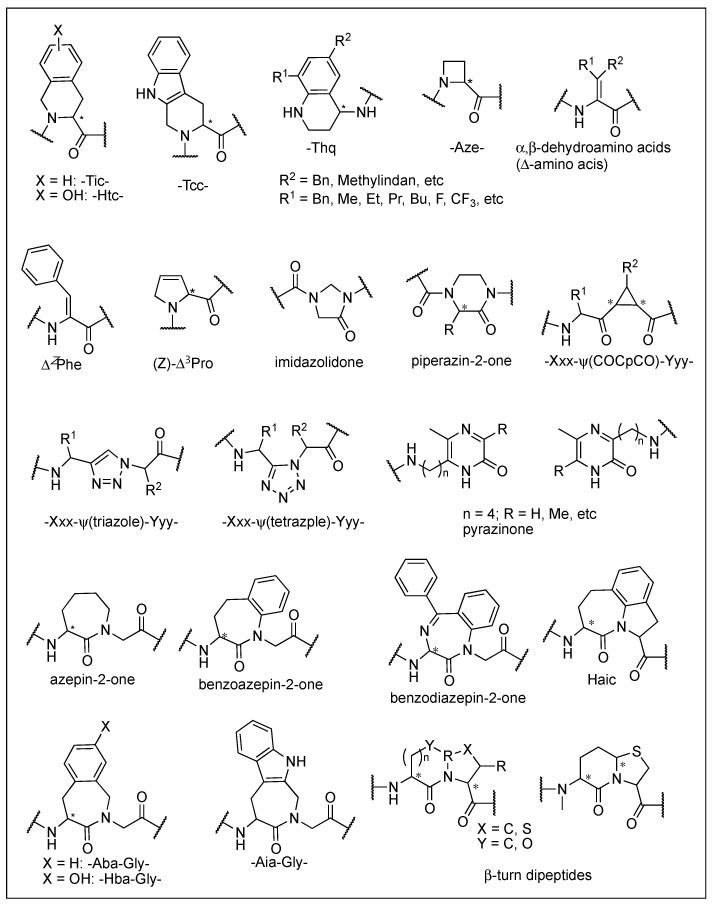
Structures of constrained amino acids and dipeptidic scaffolds. * Chiral.

Many studies have focused on restricting conformational structures to an active one using constrained amino acid analogs [266]. Tic, 7-hydroxy-1,2,3,4-tetrahydroisoquinoline-3-carboxylic acid (Htc), and β-carboline-3-carboxylic acid (Tcc) are a constrained cyclic analog of Phe, Tyr, and Trp, respectively, which are key structural elements of most opioid peptides. and Tic has been successfully applied to the design of opioid peptidomimetics (Figure 8). Dmt-Tic is the shortest peptide pharmacophore for DOR antagonism and is being widely used as a template due to the remarkable alterations in selectivity and activity [180,264,267,268]. 1,2,3,4-tetrahydroquinoline (Thq) derivative coupled with a Dmt residue to resemble JOM-13 structure showed potent MOR agonist activity [269]. Further modifications at C-6 and C-8 positions of Thq with various aromatic, cycloalkyl, heterocyclic, and alkyl groups transferred its activity to a mixed opioid activity with optimized pharmacological properties reducing tolerance and dependence as well as improving the BBB permeability [91,270,271]. The modification with 4-hydroxy 2-methylindanyl group at C-6 significantly increased efficacy at KOR (*K*_i_ = 0.77 nM, EC_50_ = 25 nM, 92% stimulation) and MOR (*K*_i_ = 0.18 nM, EC_50_ = 4.9 nM, 66% stimulation) resulting in a MOR/KOR agonist [272].

Replacement of Pro^2^ of EMs with a more constrained cyclic amino acid such as (*S*)-azetidine-2 carboxylic acid (Aze) and 3,4-dehydro-(*S*)-proline (Δ^3^Pro) improved the potency but resulted in the loss of selectivity for MOR (Figure 8) [143,273]. In nature, diverse structures of α,β-dehydroamino acids exist, and dehydrophenylalanine is the most widely used one. The substitution at position 4 of EMs retained the same high potency at MOR as β-MePhe substituted analog [273,274]. A 4-imdidazolidone moiety that contains a methylene bridge between two amide bonds was well tolerated for positions 4 and 5 of ENKs showing a similar range of affinities at MOR and DOR with a slight MOR selectivity [275]. Replacement of Gly^2^-Gly^3^-Phe^4^ moiety with an aminobutyl-substituted pyrazinone ring diminished MOR and DOR activities, whereas 6-(Dmt-NHBu)- and bis-(Dmt-NHBu)-substituted pyrazinone rings showed very potent MOR agonist and weak DOR antagonist properties [58,276,277]. Recently, triazole rings are receiving attention thanks to the simple synthetic procedure, click chemistry, and the ability to adjust flexibility on all the rotatable bonds [278]. However, the positional scanning of ENKs with a 1,4-disubstituted triazole, 1,5-disubstituted tetrazole, 4-imidazolidinone, and cyclopropane ring was not successful, with an overall loss of activity in most cases. It was suggested that natural amide bonds are critical for receptor interactions, particularly through hydrogen bondings between oxygen and hydrogen atoms [275,278,279,280]. Another positional scanning of DER-tetrapeptide with piperazin-2-one moieties, *N*,*N*′-ethylene-bridged dipeptides showed slightly different SAR depending on the chirality and backbone [281].

Bicyclic conformational constraints are the most common way of building β-turn mimics, as established by the β-turn-dipeptide. Various heterocyclic scaffolds have been applied to induce the β-turn structure of opioid peptides. Atypical MOR agonist without a cationic amino group, in which R_i_, R_i+2_, and R_i+3_ are 4-hydroxyphenethyl, phenethyl, phenethyl, respectively, was identified through combinational libraries of β-turn peptidomimetics of EM [128,139]. Another β-turn opioid peptidomimetic, thiazolidinone bicyclo [2,3]-Leu-ENK, turned out to be a weak MOR agonist/DOR antagonist [282]. Azepinone scaffold has been utilized for the formation of constrained dipeptidic moieties such as Aba, 8-hydroxy-4-amino-1,2,4,5-tetrahydro-2-benzazepin-3-one (Hba), and 4-mino-1,2,4,5-tetrahydro-indolo [2,3-c]azepin-3-one (Aia). Replacement of Tic-Gly in Dmt-Tic-Gly analogs with dipeptidic moieties, Aba-Gly and *D*Aia-Gly, shifted affinity and selectivity to afford a potent MOR agonist and DOR antagonist, respectively [283,284,285,286]. The same replacement effect was observed in DER tetrapeptide when Phe^3^-Gly^4^ was substituted by Aba-Gly [287]. Recent application of a bicyclic scaffold rendered atypical cyclic EM peptidomimetic [155]. H-Dmt-NMe-*D*Ala-[(*S*)-Aba-Gly]-NH_2_ behaves as a mixed MOR/DOR agonist with high potency (EC_50_ = 1.7 pM, 16 pM, respectively). Functional electrophysiological in vitro screening using primary cortical and spinal cord networks indicated that this was the most potent, showing potential for neuropathic pain.

##### Non-Natural Amino Acid Replacements without the Alteration of a Peptide Bond

The simplest approach to accomplish conformational changes is to replace one or several positions with non-natural amino acids, such as *D*-amino acids, α-, β-, or γ-substituted amino acids, and *N*^α^-alkylated amino acids [162]. Introduction of a *D*-amino acid alters the spatial orientation of a side chain and results in the change of the conformation of the peptide backbone. The configuration-dependent differential effects on the opioid receptor selectivity and intrinsic activity have been well evidenced [162,175]. *N*^α^-substituted amino acids also influence the conformational freedom of both the backbone and side chain of an adjacent residue and increase the peptide’s lipophilicity with the reduced number of hydrogen bondings, resulting in the enhancement of the BBB permeability. The simple introduction of a NMePhe residue at position 4 of ENK retained a good DOR activity along with the increase of lipophilicity and stability [164]. [*D*Cys^2^, NMe-*D*Cys^5^]ENK turned out to be the most potent cyclic MOR/DOR agonist (IC_50_ = 1.3, 0.02 nM in GPI and MVD, respectively), possessing a good analgesic effect [288]. FK33-824 (Tyr-*D*Ala-NMePhe-Met(O)-ol) and Metkephamid (Tyr-*D*Ala-Gly-NMeMet-NH_2_) were representative analogs showing central analgesic effects through systemic administrations, which indicate the increased BBB permeability [98].

C^α^-substituted amino acids have been used to limit the number of conformations by reducing rotational freedom around a peptide bond. The 2-aminoisobutyric acid (Aib) is a common residue to elicit a turn-structure due to the extra methyl group-induced steric hindrance [128]. Incorporation of an Aib residue into ENK (position 3) and DLT (positions 2–4) retained a similar activity at DOR without detrimental effects [167,289,290]. ICI174864 is an example showing high DOR selectivity with the Aib substitutions [174]. Substitutions of C^α,α^-dialkyl cyclic amino acids in DLT showed that the *D*-chirality of the 2nd position is not critical for the DOR selectivity, and the aromatic ring of the 3rd position is replaceable with a hydrophobic ring [172]. The replacement of Phe^3^ of DLT with azidomethyl- or piperidinylmethyl-substituted Phe residues (*S*-form) retained the same range of affinity and selectivity for DOR, whereas other derivatives lost affinities [134].

**Figure 9:**
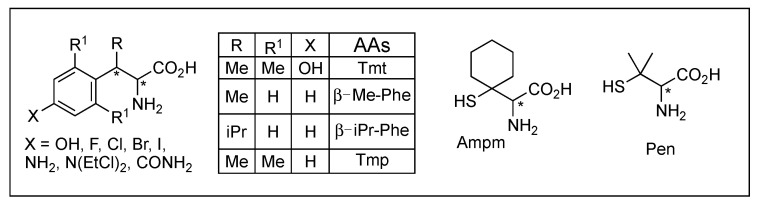
*C*^β^-substituted amino acids. * Chiral.

*C*^β^-substituted amino acids restrict the C^α^-C^β^ bond without affecting backbone angles and select one rotamer of aromatic amino acids (Figure 9) [128]. For this reason, *C*^β^-substituted amino acids have been frequently used to produce a constrained structure optimized for biological activity and physicochemical property [291]. β-MePhe, β-*i*PrPhe, β-methyl-2,6-dimethylphenylalanine, and Tmt are the most widely used in opioid peptidomimetics [292,293]. The favorable side chain rotamer for DOR was the trans form (2*S*, 3*R*), and [(2*S*, 3*R*)-β-Tmt^1^]DPDPE and [(2*S*, 3*R*)-β-*i*PrPhe^3^]DLT increased DOR selectivity with retained affinity [170,293]. *D*,*L*-amino-β-mercapto-β,β-pentamethylenepropionic acid (Apmp) has a cyclohexyl ring in place of the geminal dimethyl group of Pen, and its substitution at position 2 in DPDPE resulted in the loss of DOR affinity and selectivity due to the steric hindrance and hydrophobic property while the substitution at position 5 was tolerated [294].

Sterically constrained aromatic amino acids have been substituted for Phe and Tyr residues in opioid peptides successfully: 2′,6′-dimethylphenylalanine (Dmp), Emp, Tmp, 2′,6′-dimethyl tyrosine Dmt, and Tmt [97]. Among these, Dmt is the most successfully applied in many opioid peptides to afford [100,267]. The β-methyl group on the side chain of the amino acid basically “locks” the χ1 and χ2 conformation of the molecule [57,128]. The substitution in DPDPE and DPDPE-OMe resulted in the increase of potency at DOR and MOR but the decrease of DOR selectivity because of the enhanced MOR activity. The substituted analogs showed a significant antinociceptive activity following systemic administration [55,57]. Another trial to utilize the SAR at the MOR resulted in the discovery of a molecule with a highly balanced MOR agonist/DOR antagonist property, DIPP[ψ]-NH_2_ [100,176,177]. The substitution in DALDA resulted in the discovery of [Dmt^1^]-DALDA showing the drug-like property, long duration of antinociceptive effects in vivo after i.th. or s.c. injections, high resistance to enzymatic degradation, and slow clearance along with oral delivery potential [61,295,296,297]. Studies on the binding mode indicated that the two methyl groups on the aromatic ring of Dmt^1^ in opioid peptides participated in additional lipophilic contacts with MOR residue as well as steric influence [298]. Other constrained aromatic amino acids, Dmp, Tmp, and Emp, were introduced to the EM scaffold to lead distinct bifunctional activities such as MOR/DOR agonists or MOR agonists/DOR antagonists [97]. The alteration of EM-2 activity by Dmt^1^ and alkylated Phe^3^ residues produced a dual MOR/DOR agonist activity in Dmt-Pro-Emp-Phe-NH_2_ and a mixed MOR agonist/DOR antagonist in Dmt-Pro-Tmp-Phe-NH_2_ [97]. Substitution of a cyclic EM, Dmt-c[Lys-Phe-Phe-Asp]-NH_2_, with 2′,3′, or 4′-MePhe^4^ improved the activity slightly at DOR and dramatically at MOR, and exhibited remarkable antinociceptive effects after i.c.v. administration through potent MOR and KOR agonist activities [107].

Naphtylalanine (Nal) analogs contain a more extended aromatic ring system, like a Trp residue, and possess higher π-electron density. These bulkier residues have been successfully substituted for a Phe residue in ENKs, cyclic ENK analogs, and other opioid peptides to afford potent mixed-efficacy for opioid receptors [229,299,300]. Tyr-c[*D*Orn-2-Nal-*D*Pro-Gly] was the first potent analog with mixed MOR agonist/DOR antagonist property after less potent TIPP-NH_2_ [300]. Structural analysis indicated that the enhanced MOR activities in ENK analogs came from the relatively extended structure [299,301]. A variety of *p*-substituted phenylalanine analogs were also introduced to multiple opioid peptides including ENKs, DPDPE, and DSLET as a surrogate of Tyr^1^, and (*S*)-4-carboxamidophenylalanine (Cpa) and (*S*)-4-carboxamido-2,6-dimethylphenylalanine (Cdp) exhibited the equivalent affinity for DOR compared to parent compounds [302]. The Cdp with 2.6-dimethyl group enhanced affinity for the MOR more than Cpa analog.

**Figure 10:**
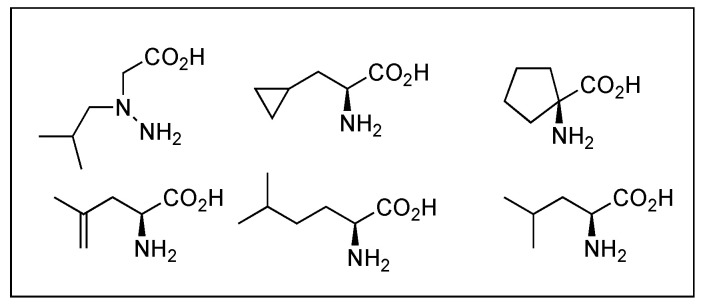
Structures of Leucine derivatives.

In most opioid peptides, the substitution of halogenated Phe residues, Phe(*p*-X), produced an enhanced opioid activity unlike other derivatives such as Phe(*p*-NH_2_), Phe(*p*-NCS), Phe(*p*-NHCOCH_2_Br) [93,303,304,305]. However, the substitution of 4-bis(2-chloroethyl)amino-*L*-phenylalanine (Mel) in Met-ENK-Arg-Phe was not tolerated at MOR and DOR, whereas *C*-terminal substitution was well tolerated at KOR [306]. The fifth residue in the address region of Leu-ENK has been modified by various analogs, including branched alkyl, cycloalkyl, or *N*^α^-*C*^α^ cyclized moiety to result in a small effect on biological activity due to the lack of role in the receptor interaction (Figure 10). Interestingly, aza-β-homoleucine or cycloleucine substituted ENK turned out to be biased with lower β-arrestin recruitment, suggesting the potential role of the 5th position in producing G-protein biased signaling [123].

Besides the modification of a backbone and the replacement of amino acid residue, a combinatorial library has also been used to identify new privileged peptidomimetic structures for opioid receptors. Acetalins (AC-RFMWMT/R/K-NH_2_) was the first potent MOR antagonists identified through the peptide library, and Tyr-*D*Nve-Gly-Nal-NH_2_ (*K*_i_ = 0.4 nM), Trp-*D*Tyr-Aba-Arg-NH_2_ (*K*_i_ = 7 nM), and *D*Phe-*D*Phe-*D*Nle-*D*Arg-NH_2_ (*K*_i_ = 1 nM) were the most potent MOR, DOR, and KOR agonists, respectively [192,307,308]. First peptidomimetic library using nonnatural oligo-*N*-substituted glycines identified a MOR ligand CHIR4531 (*K*_i_ = 6 nM) [214].

## 4. Summary and Prospective Aspects

Opioid analgesics such as morphine have been the most commonly used for severe pain despite serious side effects with long-term administration, and there are still unmet needs to solve the problems. Because the efforts to develop a new molecular entity for a therapeutic purpose have not been successful, and there are currently a decreasing number of approved drugs, it is likely important to find an alternative for the increase of productivity. In general, the lack of efficacy, toxicity, and clinical safety are the most common causes of failure in the drug discovery process. Therefore, utilization of natural opioid peptides, including EMs, DER, DLTs, ENKs, DYNs, and END, which do not possess intrinsic toxicity in the body, may provide an efficient model for novel analgesics with enhanced therapeutic advantages. In this light, opioid peptidomimetics have been studied deeply to afford applications in the therapeutic field, although the results are still insignificant: one in medical use.

Undesirable physicochemical properties and the resulting low metabolic stability and BBB permeability are the main drawbacks of opioid peptides as a drug candidate. The strategy of opioid peptidomimetic serving as a bridge between the respective characters of peptides and small molecules has solved the problems and enhanced the biological activities of natural opioid peptides as well in many cases. Mimicking natural opioid peptides is still dynamic, and there remains much room for a simplified and synthetically straightforward structure while maintaining the key structural features for the receptors. For the rational design of an opioid peptidomimetic, a full understanding of metabolic degradation pathways and SAR, and its application for pharmacokinetic and pharmacodynamic properties are significant.

Over the decades, various synthetic strategies have been developed for the preparation of non-natural amino acid derivatives or isosteres of which the substitution can modulate the intrinsic flexibility of peptide backbone. As discussed in numerous cases, opioid peptidomimetics have successfully advanced the research in opioid and pain treatment along with the advances of new technologies in structural analysis, molecular mechanics, and synthetic methodology. Systemic administrations of a few opioid peptidomimetics, including oral administration, have been shown to produce central analgesic effects through enhanced metabolic stability and BBB permeability. On the other hand, a few studies have also proven that peripherally acting opioid peptidomimetics are safe analgesic drugs with minimal side effects in the CNS. Further efforts, however, in transforming the molecules to opioid peptide-based drugs with improved safety profiles are still critical, and new formulations or routes of administration may quicken the process. Taken together, the small size of opioid peptidomimetics is likely to have a high potential of being a potent systemic and oral drug and also to provide unanticipated opportunities for the development of a novel analgesic drug that is free from opioid side effects.

## Figures and Tables

**Table 1:** Natural opioid peptides and their selectivities for the opioid receptors.

Peptides	Structure	Selectivity
β-Endorphin (END)	YGGFMTSEKSQTPLVTLFKNAIIKNAYKKGE	MOR > DOR
Enkephalins (ENKs)	YGGFL	DOR > MOR
	YGGFM	DOR > MOR
	YGGFMRF	MOR > DOR > KOR
	YGGFMRGL	MOR > DOR > KOR
Dynorphin (DYN) A	YGGFLRRIRPKLKWDNQ	KOR > MOR > DOR
DYN B	YGGFLRRQFKVVT	KOR > MOR > DOR
Endomorphin (EM)-1	YPWF-NH_2_	MOR
EM-2	YPFF-NH_2_	MOR
Dermorphines (DERs)	YaFGYPS-NH_2_	MOR
	YaFGYPK	MOR
	YaFWYPN	MOR
Deltorphine (DLT) A	YmFHLMD-NH_2_	DOR
DLT-1	YaFDVVG-NH_2_	DOR
DLT-2	YaFEVVG-NH_2_	DOR

**Table 3:** Binding affinities of potent cyclic opioid peptidomimetics at MOR, DOR, and KOR *^a^*.

Structure	*K*_i_ or IC_50_ (nM)			Ref.
	MOR	DOR	KOR	
Tyr-c^2,4^[*D*Lys-Phe-Asp]-NH_2_	0.21	461	684	[227]
Tyr-c^2,5^[*D*Lys-Phe-Phe-Asp]-NH_2_	0.35	171	1.12	[240]
Tyr-c^2,5^[*D*Cys-Phe-Phe-*D*Cys]-NH_2_	0.05	0.4	1.6	[241]
Tyr-c^2,4^[*D*Dap-Phe-Phe-Asp]-NH_2_	0.51	>1000	>1000	[227]
Tyr-c^2,5^[*cis*-*D*ACAla-Phe-Phe-Asp]-NH_2_	3.2	>1000	-	[242]
Dmt-c^2,5^[*cis*-*D*ACAla-Phe-Phe-Asp]-NH_2_	0.04	>1000	>1000	[242]
Dmt-c[*D*Lys-Phe-*D*Pro-Asp]-NH_2_ (P-317)				
Tyr-c^2,5^(-SCH_2_S-)[*D*Cys-Phe-2-Nal-Cys]-NH_2_	0.47	0.48	1.3	[229]
c^N,C^[Tyr-*D*Pro-*D*Trp-Phe-Gly]	34	-	-	[147]
Tyr-c^2,5^(-S-)[*D*Val-Gly-Phe-*D*Ala]-OH	630	0.93	1600	[223]
Tyr-c^2,5^(-S-)[*D*Ala-Gly-Phe-*D*Ala]-OH	2.0	2.0	1600	[223]
Tyr-c^2,5^(-CH_2_CH_2_-)[*D*Ala-Gly-Phe-Ala]-NH_2_	2.3	5.9	309	[225]
Tyr c^2,5^(-*cis*CH=CH-) [*D*Ala-Gly-Phe-*D*Ala]-OH	1.35	0.43	576	[224]
Tyr-c^2,5^(-NHCSNH-)[*D*Dap-Gly-Phe-Dap]-NH_2_	0.4	5.4	-	[243]
Dcp-c^2,5^[*D*Cys-Gly-Phe(4-NO_2_)-*D*Cys]-NH_2_	2.84 *^b^*	25.8	980	[244]
c^2,2′^(Tyr-*D*Cys-Gly-Phe-NH)_2_	0.60	0.87	-	[236]
c^2,2′^(Dmt-*D*Cys-Gly-Phe)_2_-cystamine	0.27	0.36	0.87	[237]
c^2,5^[*D*Asp^2^,*D*Ala^3^,Dap^5^]-Dyn A (1–11)-NH_2_	3.89	139	0.21	[195]
c^5,8^(-*trans*CH=CH-)[Ala^5^,Ala^8^]Dyn A (1–11)-NH_2_	36.0	460	2.46	[239]
c^N,5^[Trp^3^,Trp^4^,Glu^5^]-DYN A (1–11)-NH_2_	331	>8900	26.8	[238]

*^a^* The variability in the binding data (*K*_i_ or IC_50_) between various laboratories may be attributed to differences in the synaptosomal preparations, the concentration, type of radioligand, and specific activity used, and the method of reporting binding. Refer to the citations for the details. *^b^* Antagonist.
